# Elevated *α*-synuclein caused by *SNCA* gene triplication impairs neuronal differentiation and maturation in Parkinson's patient-derived induced pluripotent stem cells

**DOI:** 10.1038/cddis.2015.318

**Published:** 2015-11-26

**Authors:** L M A Oliveira, L J Falomir-Lockhart, M G Botelho, K-H Lin, P Wales, J C Koch, E Gerhardt, H Taschenberger, T F Outeiro, P Lingor, B Schüle, D J Arndt-Jovin, T M Jovin

**Affiliations:** 1 Laboratory of Cellular Dynamics, Max Planck Institute for Biophysical Chemistry, Am Fassberg 11, Göttingen, Germany; 2 Group of Membrane Biophysics, Max Planck Institute for Biophysical Chemistry, Am Fassberg 11, Göttingen, Germany; 3 Department of Neurodegeneration and Restorative Research, University Medical Center Göttingen, Waldweg 33, Göttingen, Germany; 4 Department of Neurology, University Medical Center Göttingen, Robert-Koch-Str. 40, Göttingen, Germany; 5 DFG-Research Center for Nanoscale Microscopy and Molecular Physiology of the Brain (CNMPB), Göttingen, Germany; 6 The Parkinson's Institute, 675 Almanor Ave., Sunnyvale, CA, USA

## Abstract

We have assessed the impact of *α*-synuclein overexpression on the differentiation potential and phenotypic signatures of two neural-committed induced pluripotent stem cell lines derived from a Parkinson's disease patient with a triplication of the human *SNCA* genomic locus. In parallel, comparative studies were performed on two control lines derived from healthy individuals and lines generated from the patient iPS-derived neuroprogenitor lines infected with a lentivirus incorporating a small hairpin RNA to knock down the *SNCA* mRNA. The *SNCA* triplication lines exhibited a reduced capacity to differentiate into dopaminergic or GABAergic neurons and decreased neurite outgrowth and lower neuronal activity compared with control cultures. This delayed maturation phenotype was confirmed by gene expression profiling, which revealed a significant reduction in mRNA for genes implicated in neuronal differentiation such as delta-like homolog 1 (*DLK1*), gamma-aminobutyric acid type B receptor subunit 2 (*GABABR2*), nuclear receptor related 1 protein (*NURR1*), G-protein-regulated inward-rectifier potassium channel 2 (*GIRK-2*) and tyrosine hydroxylase (*TH*). The differentiated patient cells also demonstrated increased autophagic flux when stressed with chloroquine. We conclude that a two-fold overexpression of *α*-synuclein caused by a triplication of the *SNCA* gene is sufficient to impair the differentiation of neuronal progenitor cells, a finding with implications for adult neurogenesis and Parkinson's disease progression, particularly in the context of bioenergetic dysfunction.

Parkinson's disease (PD), the second most common neurodegenerative disorder, is characterized by impairment of the motor system and associated non-motor clinical manifestations.^1^ Age^2^ and exposure to environmental toxins^3^ constitute the most important non-genetic risk factors in the development of sporadic disease. Neuronal loss is progressive, primarily (but not exclusively) dopaminergic, and accompanied by the accumulation of intracellular proteinaceous inclusions known as Lewy bodies and Lewy neurites.^4^ *α*-Synuclein (aSyn) is the main protein constituent of these inclusions^5^ and numerous findings attribute to it a central role in the pathogenesis of PD.^6, 7, 8, 9^ Both missense mutations (p.A30P, p.E46K, p.H50Q,^10^ p.G51D,^11^ p.A53T, p.A53E^12^) and increased copy number (duplication^13^ or triplication^14^) of the *SNCA* gene encoding aSyn (PARK1/4 locus) cause early onset autosomal dominant PD. In addition, multiple genome-wide association studies have established that variations at the *SNCA* locus contribute significantly to the etiology of sporadic disease.^15, 16, 17^

The induced pluripotent stem cell (iPSC) technology offers a unique and valuable tool for defining the early mechanisms underlying PD and the development of early diagnostics and new therapeutics.^18, 19, 20^ Cell lines have been generated from fibroblasts obtained from patients with a variety of neurodegenerative diseases and neurons differentiated therefrom reproduce specific features of those diseases *in vitro*.^20^ Comparisons between patient-derived and appropriately selected healthy control lines are feasible, but unfortunately phenotypic differences unrelated to the disease mechanisms arise due to the high clonal variability inherent in the generation of iPSCs and differences in the genetic background of the iPSC lines.^21, 22, 23^ Lines manipulated by single gene mutation have demonstrated the power of iPS technology for disease modeling^18, 19, 20^ with possible therapeutic potential.^24, 25^

We have examined in this study the effects of increased aSyn expression on the differentiation capacity and phenotypic signatures of two iPS clones derived from a patient with a triplication of the *SNCA* gene, and compared them with (i) lines generated by lentiviral infection of the patient cells by an shRNA construct targeting aSyn, and (ii) two control iPSC lines one from an unaffected age-matched sibling^26^ and the other from an unrelated healthy individual.^27^ All lines were differentiated by defined protocols into neurons that exhibited cardinal neuronal markers. These paradigms were used to assess differentiation capacity, cell survival, neurite outgrowth and electrophysiological properties. The results establish aSyn-dosage as an important modulator of developmental fitness of neuronal progenitor cells and support our previous findings from studies of PD patient fibroblasts^28^ and neural-committed induced pluripotent stem cells (NiPSCs) (including the knockdown lines featured in this report)^29^ exposed to toxins: (i) quantifiable reduction in viability under starvation and stress and (ii) decreased mitochondrial function and upregulated catabolism.

## Results

### Characterization and differentiation of iPSC-derived NiPSCs

NiPSC neuroprogenitor lines PI-1754 (hereafter designated SNCA_Tri, Clone 1 and Clone 2) were generated from skin biopsy of an individual with a triplication of the *SNCA* genomic locus resulting in early onset, autosomal dominant PD.^26, 29^ NiPSC line PI-1761 (control 1) was derived from the mutation-negative, unaffected sister. An unrelated NiPSC line, PI-1815-C7 (control 2), from a healthy individual was used as a secondary control. All the NiPSC lines (Supplementary Table S1) were positive for the neuronal pluripotency marker nestin and morphologically indistinguishable (Supplementary Figure S1).

NiPSCs were differentiated to dopaminergic neurons (DAn) using a 30-day two-stage protocol^28^ (see Materials and Methods; Supplementary Figure S2A). The presence of *β*-III-Tubulin and MAP2 (neuronal markers), Lmx1a and Nurr1 (key factors for dopaminergic specification) and TH (tyrosine hydroxylase, a dopaminergic marker) was confirmed in the different lines during differentiation by immunofluorescence (Supplementary Figure S2B), demonstrating that the NiPSCs were capable of generating DAn. Moreover, co-expression of TH and GIRK-2 (Supplementary Figure S2B) indicated that the DAn generated using the described protocol were mostly of the A9 subtype, the subgroup found in the *substantia nigra pars compacta* and the one most affected in PD.^30^

### *α*-Synuclein is overexpressed in the SNCA_Tri lines and further upregulated during neuronal differentiation

**Figure 1:**
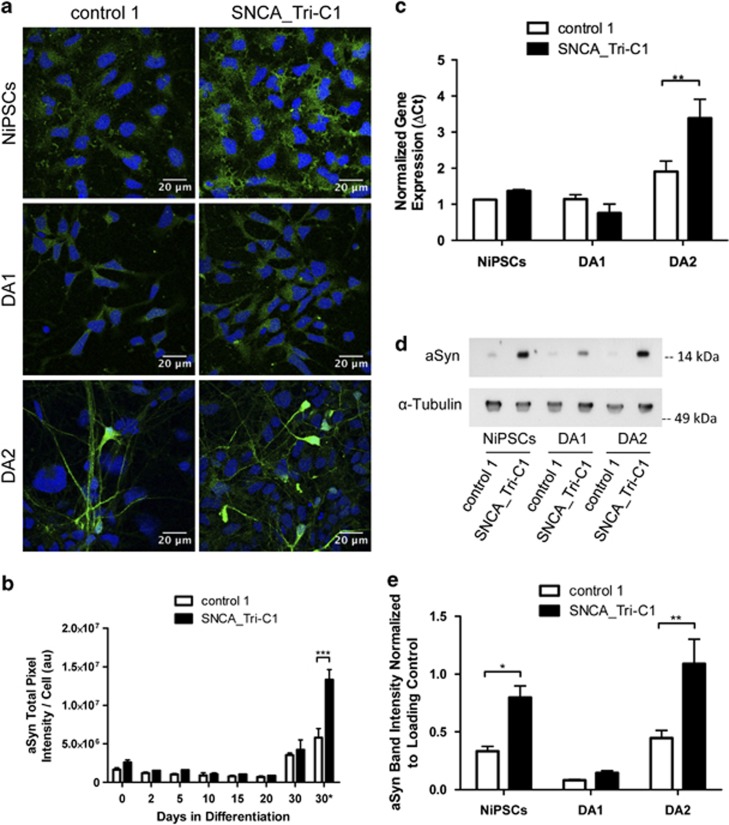
SNCA_Tri line overexpresses aSyn after differentiation. (**a**) aSyn immunofluorescence during NiPSCs differentiation (DNA counterstaining in blue). (**b**) Quantification of aSyn expression by total aSyn brightness (mean background fluorescence subtracted). The 30* time point corresponds only to cells with upregulated aSyn expression selected by image segmentation. All reported values are normalized to the number of analyzed cells. (**c**) *SNCA* mRNA abundance quantified by real-time PCR showing an increased transcription with neuronal differentiation and higher aSyn expression in the triplication line after DA2 differentiation (*n*=3). (**d**) Representative immunoblot showing greater abundance of the 15 kDa monomeric aSyn in SNCA_Tri NiPSCs and differentiated cells compared with control 1. (**e**) aSyn quantification analysis from immunoblots (*n*=4). Statistical differences were determined by *P*-values of *P<0.05, **P<0.01 and ***P<0.001

*SNCA* gene triplication has been associated with increased expression and aggregation of aSyn in PD patients^31^ and increased levels of aSyn in NiPSCs from the SNCA_Tri lines compared with those of control individuals.^29^ We quantified the differences in protein content by immunofluorescence of aSyn between patient and control lines at different stages of differentiation (Figures 1a and b). Greater integrated signal intensities (per cell) were observed in the undifferentiated state of SNC*A*_Tri and the difference (∼2-fold) was even more pronounced after differentiation (Figure 1b). aSyn expression levels were further quantified by qRT-PCR and immunoblot analysis (Figures 1c–e), confirming upregulation of mRNA in the patient lines. Although neither technique assessed heterogeneity in cellular expression, the average mRNA abundance and the immunoblot band intensities corresponding to the 15 kDa monomeric aSyn in the SNCA_Tri differentiated cells were 1.8 × and 2.4 × that of the two control lines. Surprisingly, the aSyn protein levels detected both by immunofluorescence and immunoblotting with several anti-aSyn antibodies were depressed during DA1 differentiation in the presence of fibroblast growth factor 8 (FGF8) and smoothened agonist (SAG). However, both control and SNCA_Tri showed the expected upregulation in aSyn mRNA and protein accumulation after DA2 differentiation to a neuronal phenotype.^32^

### aSyn overexpression in SNCA_Tri impairs neuronal differentiation and maturation

The overexpression of aSyn due to *SNCA* gene triplication significantly affected neuronal progenitor cells during differentiation and maturation. Upon 10 days in culture in the presence of midbrain specification factors FGF8 and SAG (DA1 medium) cell polarity became evident and cells from the patient and control lines initiated the generation of neurites positive for *β-*III-Tubulin and MAP2 (early neuronal markers). There were no perceptible differences in morphology or levels of expression of early neuronal markers between the lines (Supplementary Figure S3). However, after re-plating the cells at a density of 300 cells/mm^2^ for the second stage of differentiation in the presence of cyclic adenosine monophosphate (cAMP), brain-derived neurotrophic factor and glial cell-derived neurotrophic factor (GDNF) (DA2 medium), clear differences in neuronal development were observed (Supplementary Movie 1). The cells from the control line rapidly became post-mitotic and by day 9 in DA2, a complex neuronal network with pronounced neurite interconnectivity was evident. In contrast, the cells from the SNCA_Tri line showed a tendency to migrate and form local clusters during the first days of DA2 medium cultivation (see day 4 in Supplementary Movie 1), after which some cells continued to proliferate while those with an incipient neuronal appearance failed to develop a complex network.

**Figure 2:**
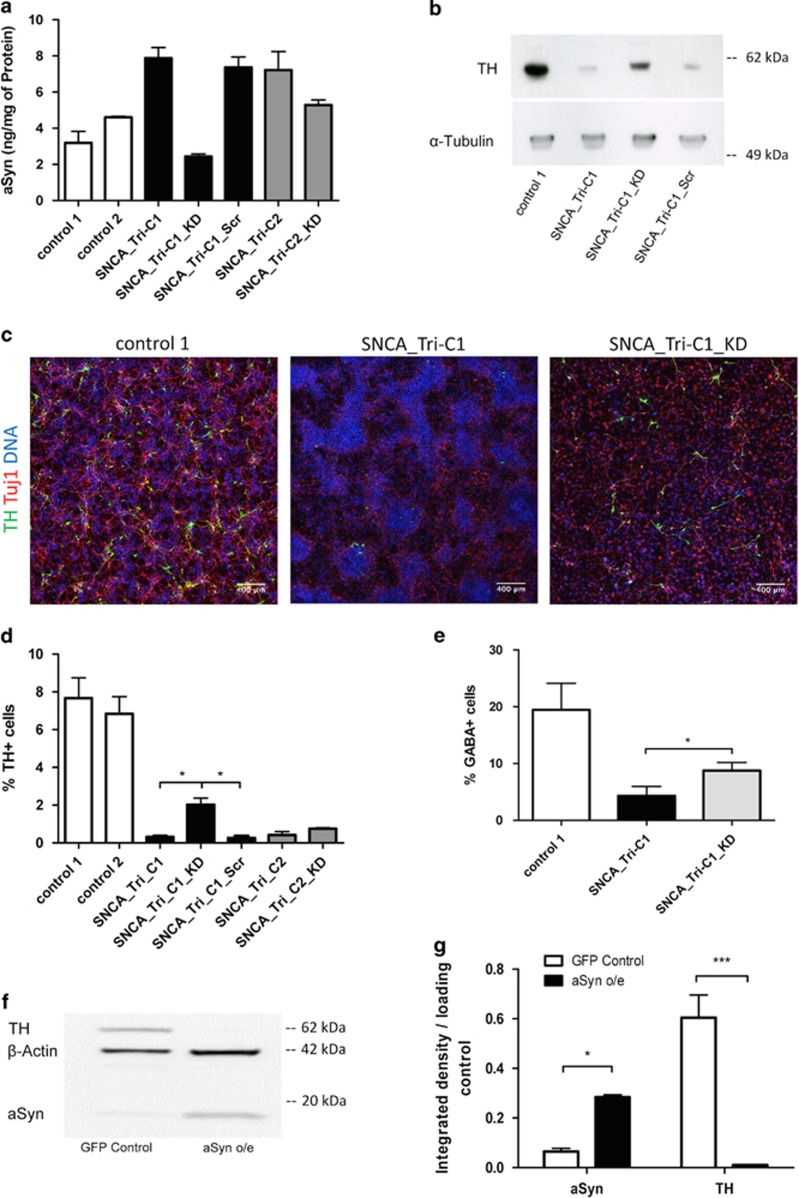
*SNCA* triplication impairs neuronal stem cell differentiation. (**a**) aSyn concentration determined by ELISA in protein extracts from NiPSCs. (**b**) Expression of tyrosine hydroxylase (TH) determined by immunoblot after DA2 differentiation. (**c**) TH immunofluorescence (green) in differentiated DAn neurons. DNA counterstained with DRAQ5 (blue); Tuj1 is represented in red (representative images from control 2, SNCA_Tri-C1-Scr, SNCA_Tri-C2 and SNCA_Tri-C2_KD lines are given in Supplementary Figure S4). (**d**) Abundance of TH^+^ neurons expressed as a percentage of the total number of cells. Neurons were obtained from three independent differentiations and imaged at a magnification of × 20. More than 15 000 cells were analyzed in each cell line. (**e**) Abundance of GABA^+^ neurons expressed as a percentage of the total number of cells. Results were acquired and analyzed as described for TH^+^ cells. (**f**) Immunoblot showing aSyn and TH expression in LUHMES cells flow-sorted for expression of aSyn-IRES-GFP (aSyn o/e) or GFP only (GFP Control). (**g**) TH quantification analysis of LUHMES immunoblots (*n*=3). Statistical differences were determined by *P*-values of *P<0.05 and ***P<0.001

To focus on the influence of aSyn on the neuronal differentiation of the patient lines, we neutralized the effects of other genomic variability by generating RNAi *SNCA* knockdowns from the two clones of SNCA_Tri using transduction with a lentivirus pLKO.1 puro vector containing an shRNA against aSyn mRNA. Infected cells (SNCA_Tri-C1_KD and SNCA_Tri-C2_KD) were continuously selected with puromycin and showed lower levels of aSyn compared with non-targeting shRNA control (SNCA_Tri-C1_Scr) cells, which demonstrated unaltered aSyn levels (Figure 2a).

The different lines were subjected to the differentiation protocol in parallel and examined for expression of TH by immunoblot and immunofluorescence (Figures 2b and c; Supplementary Figure S4). TH^+^ cells appeared in all the lines, but TH^+^ cell numbers were significantly lower in the SNCA_Tri lines (TH^+^ cells in percentage of all cells: control 1: 8% control 2: 7% SNCA_Tri-C1: 0.3% and SNCA_Tri-C2: 0.4%). Knock down of aSyn resulted in a partial recovery of the dopaminergic phenotype (2% in SNCA_Tri-C1_KD and 0.8% in SNCA_Tri-C2_KD) to a degree dependent on the knockdown level in the two cell lines, whereas no effect was observed with the non-targeted shRNA control (0.3%) (Figure 2d).

Attempts to upregulate aSyn in the control line by lentivirus transduction produced highly vulnerable cells with much higher levels of aSyn than the patient line, and which died when subjected to the differentiation protocol. To overcome this limitation, we used LUHMES cells that can be differentiated rapidly and efficiently to biochemically mature dopaminergic-like neurons.^33, 34, 35^ LUHMES cells were infected with lentiviral vectors containing aSyn with an IRES-GFP or GFP only (serving as a control). GFP-expressing cells were selected by fluorescence-activated cell sorting, differentiated and examined for TH expression by immunoblotting (Figure 2f). The aSyn levels in the aSyn overexpressing LUHMES were increased ~4.5-fold compared with the GFP-infected control LUHMES line, while TH levels were reduced more than 50-fold (Figures 2f and g), providing further evidence that overexpression of aSyn by either viral transduction or chromosomal triplication impairs neuronal differentiation.

We assessed whether the observed effects on differentiation were specific to the dopaminergic pathway by quantitating the fraction of GABA-positive cells after differentiation. Differentiated NiPSCs immunofluorescently labeled for GABA revealed values of 15% for control 1, 6.5% for SNCA_Tri and a recovery to 10% for SNCA_Tri_KD (Figure 2e), demonstrating that the impairment in differentiation on the triplication line and recovery upon aSyn knockdown are neither specific nor restricted to the dopaminergic pathway.

### Electrophysiological impairment due to aSyn overexpression in SNCA_Tri

**Figure 3:**
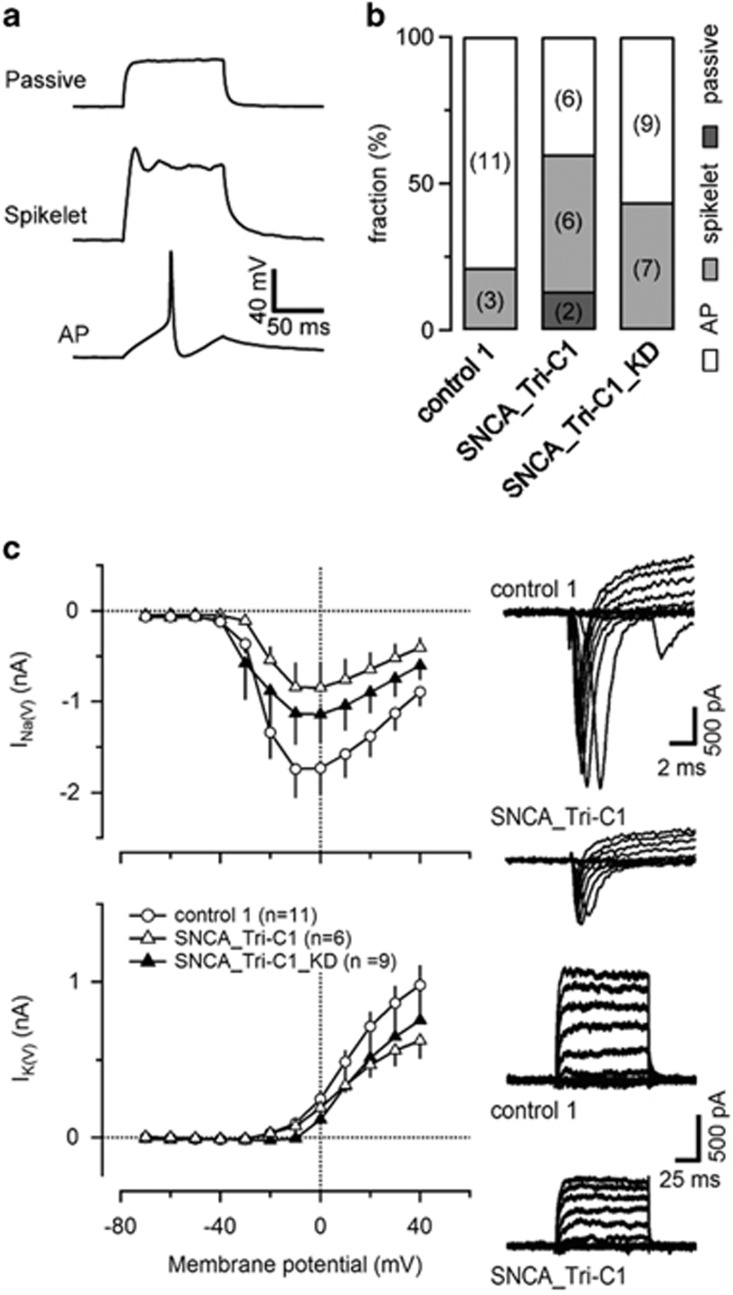
Electrophysiological characterization of differentiated NiPSCs. (**a**) Examples of three distinctive changes in membrane potential elicited by current injection. AP-firing cells were distinguished from Spikelet-firing cells by two criteria: (i) the slower kinetics of their APs measured under current clamp and (ii) the larger peak amplitudes of their *I*_Na(V)_ measured under voltage clamp. Maximum rates of rise of APs, which were obtained from the differentiated membrane potential, were generally ≥5 mV/ms for AP-firing but lower for Spikelet-firing cells. Peak amplitudes of *I*_Na(V)_ were generally ≥200 pA in AP-firing but lower in Spikelet-firing cells. In passive cells *I*_Na(V)_ was undetectable. (**b**) Relative fraction of *AP-firing*, *Spikelet-firing* and *Passive* cells. (**c**) Characterization of *I*_Na(V)_ and *I*_K(V)_ in control, SNCA_Tri and SNCA_Tri_KD cells. The mean *I–V* relationships of *I*_Na(V)_ and *I*_K(V)_ are shown in the left panels. Representative recordings are shown in the right panels for control and triplication lines. See text for details

We characterized the membrane properties of the differentiated lines by single-cell electrophysiology (Figure 3; Supplementary Figure S5). Data obtained from the cells could be categorized into three distinctive classes according to the action potential (AP)-firing pattern and amplitudes of the *I*_Na(V)_: cells that fired well-defined APs (termed *AP-firing* cells); cells that generated small and significantly slower APs (termed *Spikelet-firing* cells); and cells that were not able to generate APs in response to current injection (termed *Passive* cells) (Figure 3a). The fraction of *AP-firing* control 1 cells was 79% and 43% in the *SNCA*_Tri-C1. Interestingly, when aSyn was knocked down at the NiPSC stage, the fraction of *AP-firing* cells increased to 56%. *Passive* cells were only observed with SNCA_Tri (Figure 3b).

A more detailed summary of the properties of the *AP-firing* cells is given in Supplementary Figure S5. The absolute peak amplitudes of the APs for the SNCA_Tri line were lower compared with control; the knock down of aSyn did not exhibit a statistically significant recovery.

Voltage-gated ion conductances were also characterized. Representative recordings of *I*_Na(V)_ and *I*_K(V)_ are shown in the right panels of Figure 3c. The peak of the *I–V* curve of *I*_Na(V)_ was ∼−10 mV and the average amplitude of the *I*_Na(V)_ measured at this membrane potential was −1.7±0.3 nA for control 1 and −0.7±0.3 nA for SNCA_Tri. aSyn knockdown in the patient line rescued the *I*_Na(V)_ average amplitude by −50% (−1.1±0.4 nA). A similar tendency was observed for *I*_K(V)_ in the three groups (Figure 3c): smaller *I*_K(V)_ in SNCA_Tri when compared with control and a partial rescue of the *I*_K(V)_ amplitudes in the SCNA_Tri_KD.

### SNCA_Tri shows reduced expression of genes associated with signal transduction and neuronal differentiation

We compared the gene expression profiles of several genes associated with PD in the differentiated cells using a commercial PCR expression array. Of the 84 genes on the PCR array, 11% showed a statistically significant difference (*P*<0.05) between the SNCA_Tri and both the control and SNCA_Tri_KD lines (Table 1). Four of these were downregulated in the SNCA_Tri line and represent genes directly related to neuronal cell differentiation (*DLK, GABABR2, NURR1* and *TH*). In addition *GIRK-2* was also downregulated in SNCA_Tri, consistent with the lower levels of potassium currents observed by single-cell electrophysiology and reflecting the impaired neuronal maturation of SNCA_Tri line. The complete PD PCR-array gene expression profiles of SNCA_Tri *versus* control or SNCA_x3_KD are presented in Supplementary Table S2. Interestingly, no significant differences were observed in the expression levels of proapoptotic genes, indicating that the observed impaired differentiation is not caused by a selective neuronal death in the patient lines. We performed an LDH release assay on all lines without added toxic stress in the NiPSC state and after differentiation to assess cell fragility (Table 2). The measured LDH release was low and similar in the different lines tested in both stages, although all lines showed an increased LDH release after differentiation.

### aSyn overexpression in *SNCA*_Tri reduces neurite outgrowth

**Figure 4:**
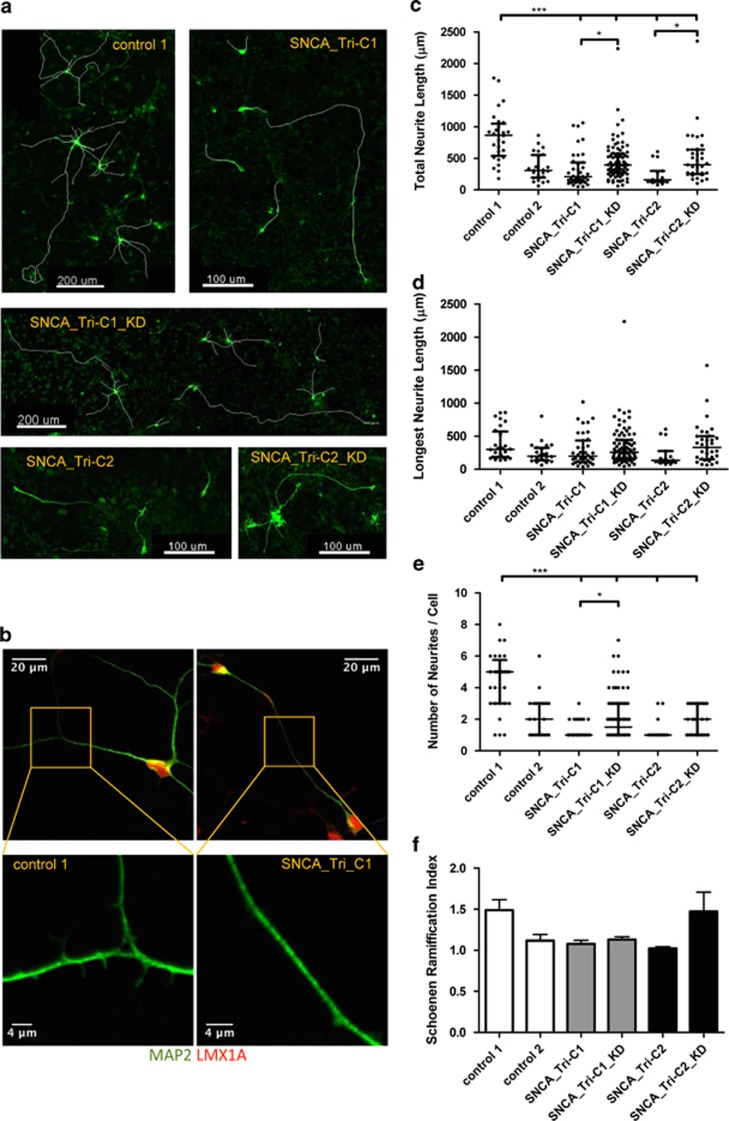
*SNCA* triplication reduces neurite outgrowth. (**a**) Neurite morphology of dopaminergic neurons from control 1, SNCA_Tri-C1, SNCA_Tri-C1_KD, SNCA_Tri_C2, and SNCA_Tri-C2_KD differentiated NiPSCs, identified by TH immunocytochemistry. (**b**) High magnification images of neurite morphology in the patient and control lines: MAP2 (green) and Lmx1A (red) (**c–f**) Quantification of dopaminergic neurite morphology per cell in terms total neurite length (**c**), mean length of the longest neurite (**d**), number of neurites (**e**) and Schoenen Ramification Index (**f**). The data are shown in scatter box plots representing the median and interquartile range of each group and explicitly explained in the Results. Statistical differences were determined by *P*-values of *P<0.05, **P<0.01 and ***P<0.001

The images in Supplementary Movie 1 demonstrate that the SNCA_Tri line failed to develop a complex neuronal network during differentiation. To further investigate the impact of the aSyn overexpression on neurite outgrowth, differentiated NiPSCs were stained for TH and neurites were traced (Figure 4a). The higher neuronal connectivity and spine formation present in control compared with the SNCA_Tri cells was perceptible at high magnification (Figure 4b). Tracing analysis showed a significant recovery upon aSyn knockdown in the number of neurites per cell and in the total neurite length (Figures 4c and e), confirming that increased aSyn gene expression had a negative impact on the neuronal development of the SNCA_Tri line. Total DAn neurite length per cell increased from 208 *μ*m in the SNCA_Tri-C1 to 394 *μ*m in the SNCA_Tri-C1_KD and from 158 *μ*m in the SNCA_Tri-C2 to 395 *μ*m in the SNCA_Tri-C2_KD (controls: 864 and 306 *μ*m) (Figure 4c); and the median number of neurites per cell increased from 1 in the patient clones to 2 after aSyn knockdown (control 1: 5; control 2: 2 neurites per cell) (Figure 4e). Interestingly, the mean length of the longest neurite (Figure 4d) and the total mean neurite length (not shown) were indistinguishable for all the lines. We attribute the reduced number of neurites in control 2 *versus* control 1 to the greater age of the control 2 individual compared with the others (Supplementary Table S1). We also determined the ratio of the maximum number of branches to the number of primary neurites, the Schoenen Ramification Index^36^ (Figure 4f), an estimate of the degree of arborization of a single neuron. No statistically significant differences were observed between the lines. Thus, the significant rescue of the number of neurites and total neurite length upon aSyn knockdown constitutes primary evidence that overexpression of aSyn significantly impairs neurite outgrowth in the NiPSCs-derived neurons.

All neurite outgrowth measurements were performed on cells that had been induced to differentiate for a total of 30 days. To rule out the possibility that the SNCA_Tri line required a longer time to develop its neuronal network than the control line, the time of differentiation was extended to a total of 64 and 75 days. Extensive interconnected neurons were evident in the control line whereas a lower and less robust neurite connectivity appeared in the SNCA_Tri line (Supplementary Figure S6).

### aSyn overexpression in SNCA_Tri leads to increased autophagic flux

**Figure 5:**
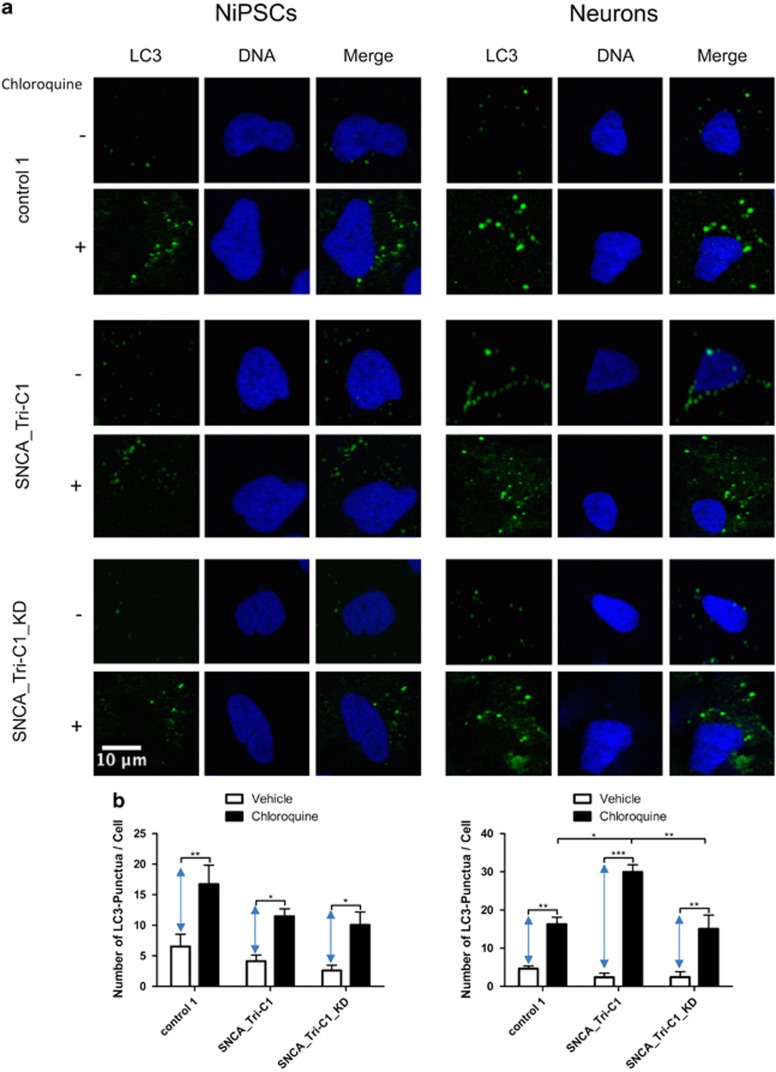
*SNCA* triplication increases macroautophagy in differentiated neurons. NiPSCs and differentiated neurons pretreated with vehicle or chloroquine (5 *μ*M) for 18 h prior to fixation and immunofluorescence analysis. (**a**) Macroautophagy flux determined by LC3 immunostaining (green) in the presence (+) or absence (−) of chloroquine. DNA was counterstained with DRAQ5 (blue). (**b**) Quantification of the intraneuronal LC3+ puncta per cell (>50 cells per line analyzed in the NiPSCs stage and >100 cells in the DA2 stage). Blue arrows designate the differences in LC3+ autophagosomes between the cells pretreated with chloroquine and vehicle, and are used as an estimation of the macroautophagic flux. Statistical differences were determined by *P*-values of *P<0.05, **P<0.01 and ***P<0.001

Impaired neurite outgrowth has been associated recently with dysregulation of autophagy.^37, 38^ To investigate whether aSyn accumulation in the SNCA_Tri line directly impacts macroautophagic flux, we determined the numbers of LC3^+^ autophagosomes in cells treated with chloroquine, a known blocker of autophagy that inhibits the fusion between autophagosomes and lysosomes.^39^ The difference between LC3 puncta in the presence and absence of chloroquine provides an estimation of the autophagic flux. Higher activity leads to a greater accumulation of LC3-II after chloroquine treatment.^29^ Counts of LC3^+^ puncta showed that the autophagic flux was comparable between the different cell lines in the NiPSCs stage. However, after differentiation and the accompanying upregulation of aSyn, the SNCA_Tri line showed increased macroautophagy compared with both the control and SNCA_Tri_KD lines (Figures 5a and b).

## Discussion

We have investigated NiPSC lines derived from a PD patient with a triplication of the *SNCA* locus and found a negative impact of increased expression of human WT aSyn on neuronal differentiation. This determination was made by comparison of the patient-derived SNCA_Tri line (2 clones) with KD lines generated by knocking down aSyn in the SNCA_Tri with a lentivirus shRNA vector, thereby circumventing phenotypic differences emerging from genetic variability when comparing different lines. A line derived from an age-matched healthy sibling was used as a standard control and some experiments were augmented by an additional control line from an older healthy individual lacking a known PD mutation.

We recently reported an increased stress vulnerability of NiPSCs from the two clones of SNCA_Tri compared with SNCA_Tri_KD_C1 and controls 1 and 2 used in this study.^29^ Although under high-glucose growth conditions the lines showed little difference in viability, upon starvation or treatment with toxicants the NiPSCs from the two SNCA_Tri clones displayed significantly increased apoptosis, delayed protein import to organelles and reduced catabolism of aggregated proteins compared with the control lines. These data indicate that the increased expression of aSyn in the patient lines resulted in neuronal precursor cells with reduced metabolic plasticity and reduced energy capacity. The focus of the present study was to determine how these lines were able to cope with the challenges posed by differentiation, a process of extensive cell remodeling that requires robust catabolic activity and ample energy reserves.

Both the control and SNCA_Tri lines showed the presence of the early neuronal markers Tuj1 and MAP2, and key factors for dopaminergic specification such as Lmx1a, Nurr1 and TH. However, the differentiation efficacy of the two independent SNCA_Tri clones was dramatically lower compared with the control lines, a result that corroborates the report that viral overexpression of aSyn in human neural progenitor cells leads to decreased levels of TH^+^ and GABA^+^ neurons.^40^ Knockdown of aSyn in both SNCA_Tri lines resulted in a rescue of the efficiency of differentiation in both lines, which correlated with the level of knockdown. The inherent clonal variability between different lines resulting from epigenetic reprogramming, which we and others^22, 41^ have observed, may result in significant differences in iPS differentiation efficiencies. Other factors related to gene expression regulating cell fate and differentiation arising from different genetic backgrounds may also compensate the impact of elevated aSyn inasmuch as the gene triplication event in the patients was not restricted to the *SNCA* locus. In the patient featured in this study, the SNCA triplication event corresponded to a 1.61–2.04 Mb region of chromosome 4 with at least 17 annotated genes.^42^ Nonetheless, the significant recovery after aSyn knockdown suggests the existence of a direct correlation between aSyn accumulation and neuronal differentiation. This conclusion is supported by the finding that overexpression of WT aSyn in LUHMES cells also significantly reduced TH levels after differentiation. Taken together, the results indicate a negative effect of aSyn overexpression and pathological accumulation on neuronal commitment and differentiation.

A possible feature leading to impaired neuronal differentiation of the SNCA_Tri line is aSyn-induced transcriptional dysregulation of genes involved in neuronal development. We compared SNCA_Tri-C1 to its knockdown line and control 1 in order to reveal the consequences of increased aSyn expression and minimize the effects of genetic differences between the siblings. The PD expression array revealed changes in nine genes, including downregulation of four genes intimately related to neuronal differentiation and specification: *NURR1*, *DLK1*, *TH*, *GABBR2* and *GIRK-2*. Nurr1 is critical for the development and maintenance of midbrain neurons. Thus, loss of Nurr1 function early during development in mice leads to the absence of midbrain neurons, while reduction of Nurr1 function in adulthood leads to a slowly progressive loss of striatal dopamine and markers of DAn.^43^ Furthermore, overexpression of aSyn in nigral DAn in rats is correlated with reduced expression of Nurr1 and its downstream targets.^44^ DLK1 is a member of the Delta/Notch protein family and participates in the specification of ventral midbrain DAn.^45^ DLK1 treatment during expansion of progenitor cells resulted in increased neurons expressing TH.^45^ Interestingly, DLK1 has also been reported to be a Nurr1 downstream target.^46^ Another gene shown to be downregulated in the triplication line is the potassium channel GIRK-2, supporting our electrophysiology results and also pointing to reduced neuronal maturation in the patient line.

Markedly different electrophysiological profiles of the various cell lines were observed by a single-cell patch-clamp. Passive, unresponsive cells were only found in the SCNA_Tri line, whereas the control and SNCA_Tri_KD lines showed higher numbers of AP-firing cells. In addition, *I*_Na(V)_ and *I*_K(V)_ currents were higher in the SNCA_Tri_KD and control 1 compared with SNCA_Tri. We conclude that reduced numbers of ion channels are present in differentiated SNCA_Tri, leading to smaller current amplitudes as a result of a lower neuronal maturation of SNCA_Tri. The SNCA_Tri cells had persistently less connectivity than the control cells after differentiation for 64–75 days (Supplementary Figure S6), and the loss of connectivity was already manifested at 30–35 days.

A significant phenotypic feature of *SNCA* triplication was the impairment of neurite outgrowth. Neurite tracing analysis showed that knock down of aSyn increased both the total neurite length and the number of neurites in TH^+^ neurons compared with SNCA_Tri. Interestingly, the mean neurite length and the Schoenen ramification index were comparable between all the lines, suggesting that aSyn can have a negative impact on the initiation of neurite formation, but not on its elongation and branching. Previous publications report contrasting findings on the effects of aSyn on neurite outgrowth. Liu *et al.*^47^ reported that aSyn promotes neurite growth in cultured primary neurons, whereas other investigators^48, 49, 50^ have shown a negative impact of aSyn on neurite outgrowth. A fundamental difference between these studies is the choice of the cellular models used to investigate the effects of aSyn. Whereas in the first study the authors used fully developed and mature rat cortical primary neurons in culture, in which neurite outgrowth is primarily regulated by the polymerization of cytoskeleton proteins (mainly tubulin), the other studies reported aSyn-associated deficits on neurite outgrowth in the context of differentiation and maturation of newly formed neurons from progenitor cells. Recently, we showed that aSyn has deleterious effects on the neurite outgrowth of primary midbrain neurons^48^ and in another study it was proposed that aSyn might modulate neurite outgrowth by negatively affecting spectrin beta non-erythrocyte 1, a protein that promotes neurite formation in differentiating SH-SY5Y cells.^51^

Reduction of neurite outgrowth can occur with upregulation of autophagy.^6, 7^ Previous publications have reported contradictory findings on the effects of aSyn accumulation on this process.^52^ The apparent discrepancies in the literature may reflect the inherent staged,^53^ inverse^54^ correlation between disease progression and protein quality control, and also the particular model systems employed, which vary in aSyn expression, post-translational modification and the kind and degree of association/aggregation. Autophagic flux was significantly increased in the SNCA_Tri line compared with both control and SNCA_Tri_KD lines after differentiation (i.e. after aSyn upregulation). Similar results were found in primary midbrain neurons, where overexpression of aSyn WT also resulted in increased autophagic flux.^48^ Upregulation of autophagy may act as a cellular compensation mechanism to clear accumulated monomeric aSyn, although it may also foster unspecific degradation of essential proteins and organelles including mitochondria, thereby contributing to the development of pathology. Under stress conditions or in the presence of proteolysis-resistant forms of aggregated aSyn, autophagy may be compromised.^55^

A number of PD-associated genes have been implicated in neuronal development, such as *SNCA*, *NURR1*, *PITX3*, *PINK1*, *LRRK2* and *VPS35*,^56^ and progenitor cells isolated from human brains with idiopathic PD appear to lack key factors required for neuronal differentiation.^57^ We propose that the differentiation deficits of the SNCA_Tri NiPSCs observed in this study may reflect functional de-differentiation (loss of function) undergone by neurons affected in and by PD over an extended period of time, whereas compensatory and neuroregenerative processes in the brain impede or at least delay the development of overtly clinical disease. The patient iPSCs were derived from a patient exhibiting clinical manifestations of PD and thus chronically exposed to the effects of *SNCA* gene triplication. In fact, a recent report found high levels of aSyn in the epidermis of PD patients but no expression in control subjects, emphasizing the systemic nature of the disease.^58^ Our results are consistent with a model in which genetic variants affecting aSyn expression modulate cellular susceptibility to neurodegeneration in PD, as has been proposed recently.^59, 60^ We also note that the procedures required to achieve differentiation *in vitro* necessarily involve numerous and complex epigenetic transformations, only some of which may reproduce the natural pathogenetic evolution in the disease context. Optimized, rational therapeutic regimens, which to date are essentially empirical, need to address both the underlying cellular processes at the molecular level^35, 61^ as well as the systemic mechanisms operating within the complex environment of the central nervous system. Of direct relevance in this connection is the recent identification of elevated mitochondrial bioenergetics (higher basal level of oxidative phosphorolyation, greater axonal mitochondrial density) and high complexity of axonal arborization as major contributors to the hightened vulnerability of nigral dopamine neurons to toxic agents.^62^ The neuroenergetic issues highlighted by the present and related studies also lend support for a major role of metabolic reprograming in neuropathogenesis, proposed by Demetrius *et al.* (reviewed in Demetrius *et al.*^63^) as an alternative to the amyloid hypothesis in the context of Alzheimer's disease and invoked here for PD. It is postulated that age-induced mitochondrial dysfunction leads to an upregulation of the complementary metabolic processes of oxidative phosphorylation (inverse Warburg effect) in neuronal mitochondria and of glycolytic activity (Warburg effect) in the cytoplasm of astrocytes. The short- and long-range neuron–astrocyte cytoarchitecture is envisioned as key in mediating both supportive as well as competitive interactions between disease-affected and normal neurons and astrocytes. Mutation or overexpression of aSyn – as in the case of the patient NiPSCs of this study and/or the result of reduced degradation – can thus be regarded as factors initially exacerbating age-related mitochondrial dysregulation at the level of individual neurons,^64^ and subsequently promoting disease progression by virtue of resource depletion (a process of ‘natural selection'^63^) engendered by the pathological neurons.

## Materials and Methods

### Generation and differentiation of patient-derived iPSCs

Fibroblast biopsies from a patient with early onset PD carrying a triplication of the SNCA locus and a four-year older mutation-negative healthy female sibling were used to generate iPS cells by The Parkinson's Institute.^26^ Recently published protocols combining neuronal induction from embryoid bodies and dual SMAD inhibition combined with PSA-NCAM magnetic-bead sorting were used to generate neuroprogenitor cell lines, NiPSC.^27^ NiPSCs were seeded at 5 × 10^2^ cells/mm^2^ on culture dishes coated with Geltrex (Life Technologies, Darmstadt, Germany, #A1413302), and propagated in NiPSCs growth medium: MACS Neuro Medium (Miltenyi Biotech, Bergisch Gladbach, Germany, #130-093-570), 1 × MACS NeuroBrew-21 (Miltenyi Biotech #130-093-566), 2 mM l-glutamine (Life Technologies #25030024), 1 × NEAA (Life Technologies #111400), 50 U/ml penicillin/streptomycin (Life Technologies #15140122), 5000 U/ml ESGRO (LIF) (Merck-Millipore, Darmstadt, Germany, #ESG1106) and 20 ng/ml bFGF (PeproTech, Hamburg, Germany, #AF-100-18B-50) for up to 25 passages. The cells were passaged at confluency with Accutase (Life Technologies #A1110501) and reseeded in a new dish for propagation or differentiation.

Differentiation into DAn was performed in a two-step process, in which 5 × 10^2^ cells/mm^2^ were first incubated for 10 days in DA1 medium: MACS Neuro Medium, 1 × MACS NeuroBrew-21, 2 mM l-glutamine 1 × NEAA, 50 U/ml penicillin/streptomycin, recombinant human-FGF-8a (R&D Systems, Wiesbaden-Nordenstadt, Germany, #4745-F8) and 1 *μ*M smoothened agonist SAG (Merck-Millipore #566660). At 10–11 days cells were reseeded at 8–10 × 10^2^ cells/mm^2^ and incubated for a minimum period of at least 20 days in the second differentiation medium, DA2: MACS Neuro Medium, 1 × MACS NeuroBrew-21, 2 mM l-glutamine 1 × NEAA, 50 U/ml penicillin/streptomycin, 20 ng/ml recombinant human GDNF (Peprotech, Rocky Hill, NJ, USA, #450-10), 25 ng/ml recombinant human-BNDF (R&D Systems #248-BD) and 1 mM dibutyryl-cAMP (Sigma Aldrich, Taufkirchen, Germany, #D0627).

For immunofluorescence microscopy and electrophysiology, cells were seeded on 10 or 12 mm diameter coverslips previously coated with 20 *μ*g/ml poly-l-lysine (Sigma Aldrich #P7890) and 20 *μ*g/ml laminin (Sigma Aldrich #L2020).

### Culture and Differentiation of LUHMES cells

Proliferating LUHMES cells were maintained and differentiated according to Scholz *et al.*^35^ LUHMES cells used in this paper were selected by fluorescence-activated cell sorting for GFP expression after infection by a WP1 Lentivirus containing the human cDNA for aSyn and an IRES-GFP sequence (see below). Proliferation medium consisted of DMEM/F12 (Life Technologies #12634010), 2 mM l-glutamine (Sigma Aldrich #G7513), 1x N2 supplement (Life Technologies #17502-048) and 40 ng/ml recombinant human basic FGF (R&D Systems #4114-TC-01M). Proliferating cells were cultured to a confluence of 80%, and then dissociated with trypsin (138 mM NaCl, 5.4 mM KCl, 6.9 mM NaHCO_3_, 5.6 mM d-glucose, 0.54 mM EDTA, 0.5 g/l trypsin from porcine pancreas type IX-S; Sigma Aldrich #T-0303) and seeded into a T175 Nunclon flask at a density of 8 × 10^6^ cells/flask, containing proliferation medium. After 24 h, proliferation medium was removed and replaced with differentiation medium, consisting of DMEM/F12, 2 mM l-glutamine, 1 × N2 supplement, 1 mM dibutyryl-cAMP (Sigma Aldrich #D0627), 2.25 *μ*M tetracycline (Sigma Aldrich #T7660) and 2 ng/ml recombinant human GDNF (R&D Systems #212-GD-050). Forty-eight hours after the medium change, cells were trypsinized and seeded into Nunc 24-well plates at a density of 250 000 cells/well, in 1 ml of differentiation medium/well. At 72 h after seeding one-half of the differentiation medium was refreshed. All flasks and plates were precoated with 50 *μ*g/ml poly-l-ornithine (Sigma Aldrich #P3655) and 1 *μ*g/ml fibronectin (Sigma Aldrich #F1141) for 24 h at 37 °C and air dried prior to seeding of cells.

### Immunofluorescence

Cells were fixed with 3.7% paraformaldehyde (PFA) in Tyrode's buffer at room temperature for 15 min and subsequently washed with 10 mM Tris-HCl, pH 7.4 saline buffer, to block PFA activity. Cells were then blocked in PBS with 0.1% Triton-X100 and 0.5% BSA or 2 mg/ml of donkey serum (when using donkey anti-goat secondary antibodies). The primary antibodies used are listed in Supplementary Table S3. The following secondary antibodies: Cy3-conjugated donkey anti-goat F(ab)_2_ (Dianova, Hamburg, Germany); Cy5-conjugated donkey anti-goat IgG (Abcam, Cambridge, UK); Oregon Green 488-conjugated goat anti-rabbit IgG (Life Technologies); and Alexa Fluor 546-conjugated goat anti-mouse F(ab)_2_ (Life Technologies) were all used at a 1:2000 dilution in blocking solution. Images were acquired using a Zeiss LSM 510 Meta confocal microscope, except for the TH+ cell quantifications where the images were acquired with an Andor Revolution XDi Spinning Disk Confocal System.

### Real-time PCR analysis

Total mRNA was isolated with RNeasy Mini Kit (Qiagen, Venlo, Netherlands, #74104), following the manufacturer's instructions. One microgram was used to synthetize cDNA (20 *μ*l reaction volume) using the Omniscript reverse transcriptase kit and oligo-dt primers (Qiagen, # 205111 and #79237) in a Thermo Hybaid PCR Express (Fischer Scientific, Schwerte, Germany). Real-time PCR analyses of 1 *μ*l of the cDNA products were performed in duplicate with iQ SYBR Green Supermix (Bio Rad, Munich, Germany, #170-8882) in a CFX96 Real time system Thermal cycler (Bio Rad). The primer pairs used for real-time amplification of aSyn are described in Rhinn *et al.*^59^ Primers for *HPRT*^41^ and QuantiTect Primer assay GAPDH mix (Qiagen #QT00079247) were used for normalization. Relative expression levels were calculated with subsequent ΔCT values that were assessed using the comparative CT method with subsequent ΔCT values normalized to the housekeeping genes *HPRT* and *GAPDH*.

### Gene expression profiling

One microgram of mRNA (isolated as described above) was converted to cDNA using the RT^2^ First Strand Kit (SABiosciences, Venlo, The Netherlands, # 330401). Real-time PCR analysis was performed using the RT^2^ Profiler Human Parkinson's disease PCR Array (SABiosciences #PAHS-124ZD-12) according to the manufacturer's instructions on a CFX96 Real-Time PCR system (Bio Rad). Gene of interest (GOI) CT values were normalized to the average CT value of five housekeeping genes (*β*-actin (*ACTB*), *β*-2-microglobulin (*B2M*), glyceraldehyde-3-phosphate (*GAPDH*), hypoxanthine-guanine phosphoribosyltransferase (*HPRT*) and ribosomal protein, large, P0 (*36-B4*)), and subsequent ΔCT values are expressed as fold change, 2^−^^ΔCT(SNCA_x3)^/2^−ΔCT(Control or SNCA_x3_KD)^. Fold regulation was calculated as −1/2^−ΔCT^ for all negative fold changes.

### Protein extraction and immunoblot analysis

NiPSCs and LUHMES were resuspended with Accutase or trypsin, respectively, washed with Tyrode's buffer, and lysed with buffer containing 50 mM Tris, 1% Triton, 250 mM NaCl, 5 mM EDTA supplemented with 1 mM PMSF and 1 × protease inhibitor cocktail (Roche Applied Science, Penzberg, Germany, #11873580001) for 30 min on ice. Subsequently, the extracts were centrifuged at 20 000 × *g*, at 4 °C, for 30 min. The supernatant was then collected and the protein concentration was measured with a bicinchoninic acid assay (Fischer Scientific #23227). The same amount of total protein for each cell lysate was loaded onto NuPAGE Novex System Bis-Tris 4–12% gels with MES (2-(*N*-morpholino) ethanesulfonic acid)-SDS buffer (Life Technologies # NP0336BOX and # NP0002, respectively). After electrophoresis, and transfer onto polyvinylidene difluoride membranes, specific protein bands were detected using appropriate primary and secondary antibodies (mAB anti-aSyn; 1 : 1000), rAB anti-TH (Merck-Millipore #AB152; 1 :  5000), and horseradish peroxidase goat anti-mouse (Dianova #111-036-003) and goat anti-rabbit (Dianova #111-035-003) secondary antibodies, followed by SuperSignal West Pico Chemiluminescent Substrate (Fischer Scientific #34077). The signals were recorded on X-ray films (GE Healthcare, Buckinghamshire, UK, #28906837) using a Kodak X-OMAT 2000 Processor, and quantified with the gel analysis tool of Fiji analysis software.^65^ For the loading control, the membranes were re-probed with a mAb anti-*α*-tubulin (a kind gift from Dr. Mary Osborn) or an mAB anti-*β*-actin (Ambion, Darmstadt, Germany, #AM4302, 1 : 5000). The results are expressed as a ratio of the aSyn and *α*-tubulin, or TH and *β*-actin signals. Total aSyn quantification by ELISA was performed using the Human alpha-Synuclein ELISA Kit (Lifetechnologies #KHB0061) following the manufacturer's protocol. Data obtained from the ELISA were then normalized to total protein.

### Lentivirus

*Constructs*: Full-length human aSyn cDNA (SNCA, gene association NM000345) was subcloned into a second-generation lentiviral vector pWPI (Tronolab, Lausanne, Switzerland), followed by an IRES-GFP sequence, and the original vector promoter (EF1*α*) was replaced by the chicken *β*-actin promoter. A vector containing only the IRES-GFP cassette was used for control experiments. For lentiviral shRNA production the third-generation vector pLKO.1 puro (Sigma Aldrich) containing the sequence 5′-ACCAAAGAGCAAGTGACAAAT-3′ (clone ID, TRCN0000003736) was used to knock down human aSyn gene expression. Control experiments were performed using the same vector containing the non-target shRNA sequence 5′CCTAAGGTTAAGTCGCCCTCG3′. All cloned sequences were verified by automated sequencing (StartSeq, Mainz, Germany).

*Lentivirus generation and transgene expression*: Second-generation lentiviral particles were generated as previously described.^66^ Briefly, the modified transfer-vectors were purified and co-transfected with the packaging vectors (Tronolab, Switzerland) into 293 FT cells (Life Technologies) for 48 h. The supernatant was collected, concentrated by PEG-it Virus Precipitation Solution (Systems Biosciences, Heidelberg, Germany) and resuspended in Panserin 401 (PAN, Germany). Transgene expression was determined by qRT-PCR using SYBR GREEN after infection of the 293-HEK cells using specific primers to the woodchuck hepatitis virus *post*-transcriptional regulatory element (WPRE) as described.^67^ Virus were used at the same infectivity in all applications and stored at −80 °C. NiPSCs were infected with the pLKO.1 containing the shRNA and non-target sequences at 50–70% cell confluency. Cells were washed with NiPSCs medium after 24 h and selected with 3 *μ*M puromycin after 48–72 h.

### Electrophysiology

Patch-clamp recordings were made from NiPSCs at 45 days of differentiation by using an EPC-10 amplifier controlled by Pulse software (HEKA Elektronik, Lambrecht/Pfalz, Germany). Sampling intervals and filter settings were 20 *μ*s and 4.5 kHz, respectively. Cells were visualized by differential interference contrast microscopy through a × 60 water-immersion objective (NA 1.0), (Olympus, Darmstadt, Germany) using an Axioskop FS microscope (Zeiss, Germany). All experiments were performed at room temperature. Patch pipettes were prepared from borosilicate glass (Science Products GmbH, Hofheim am Taunus, Germany) and pulled on a P-97 micropipette puller (Sutter Instrument, Novato, CA, USA) with an open tip resistance ranging from 4–6 MΩ. Pipettes were coated with dental wax to minimize fast capacitive transients during voltage-clamp experiments and to reduce stray capacitance. Access resistance (Rs) values were ≤20MΩ. Rs compensation was set to 60–70% (10 *μ*s delay) during voltage-clamp experiments. Patch pipettes were filled with a solution containing: 100 mM potassium gluconate, 60 mM KCl, 10 mM HEPES, 0.5 mM EGTA, 5 mM Na_2_-phosphocreatine, 4 mM ATP-Mg, 0.3 mM GTP, set at pH 7.3 with KOH. The standard extracellular recording saline solution contained: 125 mM NaCl, 2.5 mM KCl, 10 mM glucose, 25 mM NaHCO_3_, 1.25 mM NaH_2_PO_4_, 2 mM CaCl_2_, and 1 mM MgCl_2_, 0.4 mM ascorbic acid, 3 mM myo-inositol, and 2 mM Na-pyruvate at pH 7.3 when bubbled with carbogen (95% O_2_, 5% CO_2_).

Sodium (*I*_Na(V)_) and potassium (*I*_K(V)_) currents were evoked by step depolarizations (50 and 100 ms) from a holding potential of −70 mV with 10 mV increments up to +40 mV. Recordings with leak currents >100 pA were excluded from the analysis. APs were measured in the current-clamp mode of the EPC-10 after carefully adjusting the fast-capacitance cancellation in cell-attached mode. The membrane potential was adjusted to −70 mV in all current-clamp recordings by injecting a small hyper or depolarizing current. APs were elicited by depolarizing current injections, starting from −20 to 170 pA in 10 pA increments. In all the three cell lines, *I*_Na(V)_ was completely blocked after addition of 2 *μ*M tetrodotoxin to the bath solution (data not shown). Offline analysis was performed with ‘IgorPro' software (Wavemetrics, Portland, OR, USA).

### Cytotoxicity

Cytotoxicity was assessed in NiPSCs and differentiated neurons using the LDH cytotoxicity detection kit (Roche Applied Science #11644793001). Culture media were collected from the cells of interest (*n*=3 independent wells) and the LDH activity was determined following the manufacturer's protocol. Results are expressed as the percentage of total LDH activity determined in parallel wells after total cell lysis (*n*=3). The results were measured in a PHERAstar FS plate reader (BMG Labtech, Offenburg, Germany).

### Neurite tracing

Neurite outgrowth was assessed in DAn, which were identified by immunofluorescence using an anti-TH antibody (Supplementary Table S2). The length of each dendritic segment was determined by tracing the center of the dendritic shaft, using the Autopath method available in the Filament Tracing Tool from Imaris v7.6.1. For each neuron, the number of neurites originating from the soma, the individual length of the longest single neurite, and the total length of all neurites in one neuron, were determined. A Schoenen ramification index^36^ for each neuron was then calculated by dividing the maximum number of neurites by the number of primary neurites, i.e. the number of neurites arising directly from the soma.

### Autophagy

NiPSCs and differentiated neurons were incubated to determine the macroautophagy flux with vehicle or chloroquine (5 *μ*M) for 18 h in NiPSCs growth medium or DA2 medium, respectively, supplemented with 1 × B-27 minus anti-oxidants (Life Technologies, #10889-038) instead of 1 × MACS NeuroBrew-21, prior to fixation and immunostaining with anti- LC3 antibody (Supplementary Table S2). Confocal images were obtained at x63 magnification, segmented by thresholding to include only the LC3 signal within the autophagosomes which represents the LC3-II fluorescent component.^68^ Using Imaris analysis software, the number of LC3^+^ puncta per cell was estimated. The minimum size of each punctum was defined as 0.2 *μ*m^2^. The autophagic flux was represented as the difference of the number of LC3 puncta for each cell line in the presence and absence of chloroquine.

### Statistical analysis

Statistical analyses were performed using the GraphPad PRISM 5 software. Values are expressed as mean±S.E.M. Statistical significance was tested by either one- or two-way ANOVA with a Bonferroni post-test, except for the neurite outgrowth experiments in Figure 4, where statistical significance was tested using a one-way ANOVA with the non-parametric Kruskal–Wallis test followed by a Dunns post-test for multiple comparisons. Statistical differences were determined by *P*-values of *P<0.05, **P<0.01, and ***P<0.001.

## Figures and Tables

**Table 1:** Differential expression of Parkinson's disease-related genes in SNCA_Tri-C1, control 1 and SNCA_Tri-C1_KD lines

**Gene symbol**	**Gene name**	**Gene description**	**Fold change SNCA_Tri *versus* control 1**	***P-*****value**	**Fold change SNCA_Tri *versus* SNCA_Tri_KD**	***P***-**value**	**Function**
DLK1	DLK	Delta-like 1 homolog (Drosophila)	−1.65	0.017	−1.36	0.006	Signal transduction
GABBR2	GABABR2	γ-Aminobutyric acid B receptor	−3.60	0.022	−2.96	0.018	Signal transduction
HSPA4	HS24	Heat shock 70 kDa protein 4	1.32	0.003	1.13	0.032	Heat shock response
KCNJ6	GIRK-2	K inwardly rectifying channel	−3.17	3.1 × 10^−5^	−2.59	3.1 × 10^−4^	Ion transport
NR4A2	NURR1	Nuclear receptor subfamily 4, group A, member 2	−7.79	0.030	−3.97	0.040	Signal transduction
SNCA	PARK1	α-Synuclein	1.85	0.029	4.68	0.005	–
TH	TYH	Tyrosine hydroxylase	−10.16	8.8 × 10^−5^	−5.54	4.2 × 10^−4^	Signal transduction
TPBG	M6P1	Trophoblast glycoprotein	−2.13	0.008	−2.21	0.008	Cell adhesion
YWHAZ	YWHAD	Tyrosine 3-monooxygenase	1.07	0.005	1.18	0.003	Inflammation

**Table 2:** Cytotoxicity levels at the NiPSC stage and after differentiation (*n*=3, mean±S.E.M.)

	**NiPSCs**	**DA2**
control 1	4.2±0.4	5.9±0.7
SNCA_Tri-C1	3.2±0.3	7.5±0.4
SNCA_Tri-C1_KD	3.5±0.2	6.8±0.5

## Abbreviations

aSyn: *α*-synuclein
cAMP: cyclic adenosine monophosphate
DA1: differentiation medium 1
DA2: differentiation medium 2
DAn: dopaminergic neurons
DLK1: delta-like homolog 1
FGF8: fibroblast growth factor 8
GABABR2: gamma-aminobutyric acid type B receptor subunit 2
GDNF: glial cell-derived neurotrophic factor
GIRK-2: G-protein-regulated inward-rectifier potassium channel 2
iPSCs: induced pluripotent stem cells
LUHMES: Lund human mesencephalic cell line
NiPSCs: neural-committed induced pluripotent stem cells
Nurr1: nuclear receptor related 1 protein
PD: Parkinson's disease
SAG: smoothened agonist
*SNCA*: human *α*-synuclein gene
TH: tyrosine hydroxylase
