# Supplementary material for: Elevated α-synuclein caused by SNCA gene triplication impairs neuronal differentiation and maturation in Parkinson's patient-derived induced pluripotent stem cells
Source: Cell Death Dis. 2015 Nov 26;6(11):e1994–. doi: 10.1038/cddis.2015.318 (PMC4670926; doi:10.1038/cddis.2015.318)
Supplement: Supplementary Table S1 [file cddis2015318x1.docx]

| **Supplementary Table 1 – Designations and etiology of the lines used in this study** | | | | | | | | |  | |  |  |
| --- | --- | --- | --- | --- | --- | --- | --- | --- | --- | --- | --- | --- |
|  |  | |  |  |  |  |  |  | |  |  |  |
| **Patient ID** | | | **Clone ID** | **Alter-native ID** | **Reprogramming method** | **Age at biopsy** | **Gender** | **Disease** | |  |  |  |
| PI-1754 | | SNCA_Tri | clone 1 | C7 | retroviral integrating four factors^1^ | 42 yrs | male | clinical description^2^ | |  |  |  |
| PI-1754 | | SNCA_Tri | clone 2 | 1754-MIT | lentiviral integrating single vector containing all four factors^3^ | 42 yrs | male | clinical description^2^ | |  |  |  |
| PI-1761 | | sibling control | control 1 | C1 | retroviral integrating four factors^1^ | 46 yrs | female | healthy | |  |  |  |
| PI-1815 | | healthy control | control 2 | C7 | retroviral integrating four factors^1^ | 62 yrs | male | healthy | |  |  |  |

^1^ Takahashi K, Tanabe K, Ohnuki M, et al. Induction of pluripotent stem cells from adult human fibroblasts by defined factors. Cell 2007, **131**:861-872.

^2^ Byers B, Cord B, Nguyen HN, et al. SNCA triplication Parkinson's patient's iPSC-derived DA neurons accumulate α-synuclein and are susceptible to oxidative stress. PloS one 2011, **6**:e26159.

^3^ Somers A, Jean JC, Sommer CA, et al. Generation of transgene-free lung disease-specific human induced pluripotent stem cells using a single excisable lentiviral stem cell cassette. Stem cells 2010, **28**:1728-1740.
