# Supplementary material for: Elevated α-synuclein caused by SNCA gene triplication impairs neuronal differentiation and maturation in Parkinson's patient-derived induced pluripotent stem cells
Source: Cell Death Dis. 2015 Nov 26;6(11):e1994–. doi: 10.1038/cddis.2015.318 (PMC4670926; doi:10.1038/cddis.2015.318)
Supplement: Supplementary Table S2 [file cddis2015318x2.docx]

**Supplementary Table 2 – Full differential expression profile of Parkinson’s disease-related genes in SNCA_Tri vs both the control and SNCA_Tri_KD lines**

| **Gene**  **Symbol** | **Gene**  **Name** | **Gene Description** | **Fold Change**  **SNCA_Tri vs Control** | **p value** | **Fold Change**  **SNCA_Tri vs SNCA_Tri_KD** | **p value** | **Function** |
| --- | --- | --- | --- | --- | --- | --- | --- |
| ALDH1A1 | ALDC | Alde hyde dehydrogenase 1 family, member A1 | 1.14 | 0.295 | 1.05 | 0.637 | Oxidative Stress |
| APC | BTPS2 | Adenomatous polyposis coli | -1.19 | 0.762 | 1.60 | 0.248 | Signal Transduction |
| APP | ABETA | Amyloid beta (A4) precursor protein | 1.12 | 0.614 | 1.06 | 0.686 | Signal Transduction |
| ATP2B2 | PMCA2 | ATPase, Ca++ transporting, plasma membrane 2 | -9.29 | 0.241 | 2.02 | 0.269 | Ion Transport |
| ATXN2 | ATX2 | Ataxin 2 | 1.31 | 0.210 | 1.01 | 0.849 | Parkin Substrate |
| ATXN3 | ATX3 | Ataxin 3 | 1.26 | 0.294 | 1.30 | 0.250 | Parkin Substrate |
| BASP1 | CAP-23 | Brain abundant, membrane attached signal protein 1 | -1.30 | 0.354 | 1.96 | 0.064 | Transcription |
| BDNF | MGC34632 | Brain-derived neurotrophic factor | -1.39 | 0.424 | 2.13 | 0.090 | Anti-Apoptosis |
| CADPS | CADPS1 | Ca++-dependent secretion activator | -1.58 | 0.007 | 8.22 | 5.3x10^-5^ | Ion Transport |
| CASP1 | ICE | Caspase 1, apoptosis-related cysteine peptidase | 1.14 | 0.295 | 1.05 | 0.637 | Pro-Apoptosis |
| CASP3 | CPP32 | Caspase 3, apoptosis-related cysteine peptidase | 1.01 | 0.874 | 1.68 | 0.003 | Pro-Apoptosis |
| CASP7 | CMH-1 | Caspase 7, apoptosis-related cysteine peptidase | -1.06 | 0.435 | -1.12 | 0.151 | Mitochondria |
| CASP8 | ALPS2B | Caspase 8, apoptosis-related cysteine peptidase | 3.78 | 0.018 | -1.16 | 0.552 | Pro-Apoptosis |
| CASP9 | APAF-3 | Caspase 9, apoptosis-related cysteine peptidase | 1.28 | 0.145 | -1.16 | 0.162 | Pro-Apoptosis |
| CDC27 | ANAPC3 | Cell division cycle 27 homolog (S. cerevisiae) | 1.15 | 0.361 | 1.11 | 0.453 | Ubiquitination |
| CDC42 | CDC42Hs | Cell division cycle 42 (GTP binding protein, 25kDa) | 1.20 | 0.442 | 1.19 | 0.295 | Cytoskeleton |
| CDH8 | Nbla04261 | Cadherin 8, type 2 | -1.56 | 0.467 | 4.47 | 0.107 | Cell Adhesion |
| CHGB | SCG1 | Chromogranin B (secretogranin 1) | -1.76 | 0.027 | 2.95 | 0.018 | Secretion |
| CUL2 | MGC131970 | Cullin 2 | 1.07 | 0.622 | 1.20 | 0.274 | Ubiquitination |
| CXXC1 | CFP1 | CXXC finger protein 1 | 1.25 | 0.293 | 1.03 | 0.759 | Ion Transport |
| D4S234E | D4S234 | DNA segment on chromosome 4 (unique) | -1.12 | 0.813 | 2.52 | 0.141 | Signal Transduction |
| DDC | AADC | Dopa decarboxylase (aromatic L-amino acid decarboxylase) | -2.07 | 0.078 | 7.01 | 0.005 | Signal Transduction |
| DLK1 | DLK | Delta-like 1 homolog (Drosophila) | -1.65 | 0.017 | -1.36 | 0.006 | Signal Transduction |
| DRD2 | D2DR | Dopamine receptor D2 | -14.08 | 0.116 | 1.00 | 0.847 | Signal Transduction |
| EGLN1 | C1orf12 | Egl nine homolog 1 (C. elegans) | 1.10 | 0.504 | 1.34 | 0.100 | Ion Transport |
| FBXO9 | FBX9 | F-box protein 9 | -1.01 | 0.965 | -1.05 | 0.955 | Ubiquitination |
| FGF13 | FGF-13 | Fibroblast growth factor 13 | -2.07 | 0.002 | -1.30 | 0.109 | Signal Transduction |
| FN1 | CIG | Fibronectin 1 | 2.01 | 0.181 | -1.66 | 0.065 | Cell Adhesion |
| GABBR2 | GABABR2 | Gamma-aminobutyric acid (GABA) B receptor, 2 | -3.60 | 0.022 | -2.96 | 0.018 | Signal Transduction |
| GBE1 | GBE | Glucan (1,4-alpha-), branching enzyme 1 | 1.22 | 0.257 | 1.46 | 0.069 | Ion Transport |
| GPR37 | EDNRBL | G protein-coupled receptor 37 | 1.12 | 0.637 | -1.06 | 0.916 | Parkin Substrate |
| GRIA3 | GLUR-C | Glutamate receptor, ionotrophic, AMPA 3 | -1.57 | 0.168 | 1.08 | 0.477 | Ion Transport |
| HSPA4 | HS24 | Heat shock 70kDa protein 4 | 1.32 | 0.003 | 1.13 | 0.032 | Heat Shock Response |
| HTR2A | 5-HT2A | 5-hydroxytryptamine (serotonin) receptor 2A | 1.07 | 0.567 | 1.05 | 0.637 | Signal Transduction |
| KCNJ6 | GIRK-2 | Potassium inwardly-rectifying channel | -3.17 | 3.1x10^-5^ | -2.59 | 3.1x10^-4^ | Ion Transport |
| LRRK2 | PARK8 | Leucine-rich repeat kinase 2 | -6.09 | 0.087 | 1.16 | 0.562 | Ubiquitination |
| MAPK9 | JNK2 | Mitogen-activated protein kinase 9 | -1.10 | 0.805 | 1.16 | 0.482 | Pro-Apoptosis |
| MAPT | TAU | Microtubule-associated protein tau | -2.54 | 0.171 | 4.61 | 0.112 | Cytoskeleton |
| NCOA1 | F-SRC-1 | Nuclear receptor coactivator 1 | 1.14 | 0.512 | 1.20 | 0.404 | Transcription |
| NEFL | NF-L | Neurofilament, light polypeptide | -1.84 | 0.008 | 1.13 | 0.003 | Cytoskeleton |
| NFASC | NF | Neurofascin | -3.84 | 0.019 | 5.55 | 0.136 | Cell Adhesion |
| NR4A2 | NURR1 | Nuclear receptor subfamily 4, group A, member 2 | -7.79 | 0.030 | -3.97 | 0.040 | Signal Transduction |
| NRXN3 | MGC176711 | Neurexin 3 | -1.38 | 0.692 | 6.35 | 0.114 | Cell Adhesion |
| NSF | SKD2 | N-ethylmaleimide-sensitive factor | -1.01 | 0.997 | 1.38 | 0.073 | Ion Transport |
| NTRK2 | TRKB | Neurotrophic tyrosine kinase, receptor, type 2 | 1.08 | 0.669 | 1.24 | 0.388 | Signal Transduction |
| OPA1 | MGM1 | Optic atrophy 1 (autosomal dominant) | 1.05 | 0.703 | 1.49 | 0.084 | Anti-Apoptosis |
| PAN2 | FLJ39360 | Poly(A) specific ribonuclease subunit homolog (S. cerevisiae) | 1.07 | 0.678 | -1.12 | 0.687 | Ubiquitination |
| PARK2 | PRKN | Parkinson protein 2, E3 ubiquitin protein ligase (parkin) | -1.20 | 0.879 | 1.00 | 0.716 | Ubiquitination |
| PARK7 | DJ-1 | Parkinson protein 7 | 1.23 | 0.008 | -1.03 | 0.493 | Parkin Complex |
| PINK1 | PARK6 | PTEN induced putative kinase 1 | 1.08 | 0.738 | 1.19 | 0.477 | Mitochondria |
| PPID | CYP-40 | Peptidylprolyl isomerase D | 1.28 | 0.009 | -1.06 | 0.322 | Anti-Apoptosis |
| PRDX2 | NKEFB | Peroxiredoxin 2 | 1.29 | 0.010 | 1.08 | 0.166 | Inflammation |
| PSEN2 | AD4 | Presenilin 2 | -1.09 | 0.623 | 1.05 | 0.658 | Pro-Apoptosis |
| PTEN | PTEN1 | Phosphatase and tensin homolog | -1.03 | 0.970 | 1.33 | 0.311 | Pro-Apoptosis |
| RGS4 | RGP4 | Regulator of G-protein signaling 4 | -6.69 | 0.045 | 1.05 | 0.637 | Signal Transduction |
| RTN1 | NSP | Reticulon 1 | -8.31 | 0.165 | 2.40 | 0.141 | - |
| S100B | S100 | S100 calcium binding protein B | -2.06 | 0.362 | 2.38 | 0.276 | Ion Transport |
| 38596 | PNUTL1 | Septin 5 | -2.15 | 0.077 | 2.74 | 0.119 | Cytoskeleton |
| SKP1 | EMC19 | S-phase kinase-associated protein 1 | 1.12 | 0.608 | 1.37 | 0.227 | Ubiquitination |
| SLC18A2 | VMAT2 | Solute carrier family 18 (vesicular monoamine), member 2 | -1.22 | 0.356 | 1.12 | 0.489 | Transporters |
| SLC25A4 | AAC1 | Solute carrier family 25 (mitochondrial carrier), member 4 | -1.07 | 0.759 | 1.17 | 0.357 | Mitochondria |
| SLC6A3 | DAT | Neurotransmitter transporter, dopamine, member 3 | 1.14 | 0.295 | 1.05 | 0.637 | Signal Transduction |
| SLIT1 | SLIL1 | Slit homolog 1 (Drosophila) | -1.12 | 0.867 | 4.83 | 0.155 | Ion Transport |
| SNCA | PARK1 | Synuclein, alpha (non A4 component of amyloid precursor) | 1.85 | 0.029 | 4.68 | 0.005 | - |
| SPEN | SHARP | Spen homolog, transcriptional regulator (Drosophila) | 1.02 | 0.820 | 1.01 | 0.869 | Signal Transduction |
| SRSF7 | SFRS7 | Serine/arginine-rich splicing factor 7 | 1.06 | 0.692 | -1.18 | 0.226 | Ion Transport |
| STUB1 | UBOX1 | STIP1 homology and U-box containing protein 1 | 1.14 | 0.443 | 1.07 | 0.629 | Parkin Complex |
| SV2B | KIAA0735 | Synaptic vesicle glycoprotein 2B | -1.51 | 0.433 | 1.05 | 0.637 | Synaptic Vesicles |
| SYNGR3 | MGC20003 | Synaptogyrin 3 | -1.51 | 0.478 | 1.99 | 0.207 | Synaptic Vesicles |
| SYT1 | SVP65 | Synaptotagmin I | -1.24 | 0.890 | 1.16 | 0.566 | Synaptic Vesicles |
| SYT11 | SYT12 | Synaptotagmin XI | -2.33 | 0.008 | 1.35 | 0.249 | Synaptic Vesicles |
| TCF7L2 | TCF4 | Transcription factor 7-like 2 (T-cell specific, HMG-box) | 1.23 | 0.008 | -1.25 | 0.008 | Anti-Apoptosis |
| TH | TYH | Tyrosine hydroxylase | -10.16 | 8.8x10^-5^ | -5.54 | 4.2x10^-5^ | Signal Transduction |
| TPBG | M6P1 | Trophoblast glycoprotein | -2.13 | 0.008 | -2.21 | 0.008 | Cell Adhesion |
| UBA1 | UBA1A | Ubiquitin-like modifier activating enzyme | -1.19 | 0.189 | 1.17 | 0.231 | Ubiquitination |
| UBB | UBA52 | Ubiquitin B | 1.09 | 0.289 | 1.11 | 0.057 | Ubiquitination |
| UBE2I | UBC9 | Ubiquitin-conjugating enzyme E2I | 1.38 | 0.058 | 1.02 | 0.803 | Ubiquitination |
| UBE2K | UBC1 | Ubiquitin-conjugating enzyme E2K | 1.04 | 0.735 | 1.07 | 0.612 | Ubiquitination |
| UBE2L3 | UbcM4 | Ubiquitin-conjugating enzyme E2L 3 | 1.32 | 0.095 | -1.01 | 0.953 | Ubiquitination |
| UCHL1 | PARK5 | Ubiquitin carboxyl-terminal esterase L1 | -1.90 | 0.033 | 1.06 | 0626 | Ubiquitination |
| USP34 | FLJ43910 | Ubiquitin specific peptidase 34 | 1.03 | 0.766 | -1.08 | 0.954 | Ubiquitination |
| VAMP1 | VAMP-1 | Vesicle-associated membrane protein 1 (synaptobrevin 1) | -1.04 | 0.999 | -1.42 | 0.171 | Mitochondria |
| VDAC3 | HD-VDAC3 | Voltage-dependent anion channel 3 | -1.10 | 0.727 | 1.02 | 0.798 | Ion Transport |
| YWHAZ | YWHAD | Tyrosine 3-monooxygenase activation protein | 1.07 | 0.005 | 1.18 | 0.003 | Inflammation |
