# Supplementary material for: Elevated α-synuclein caused by SNCA gene triplication impairs neuronal differentiation and maturation in Parkinson's patient-derived induced pluripotent stem cells
Source: Cell Death Dis. 2015 Nov 26;6(11):e1994–. doi: 10.1038/cddis.2015.318 (PMC4670926; doi:10.1038/cddis.2015.318)
Supplement: Supplementary Table S3 [file cddis2015318x3.docx]

**Supplementary Table 3 - Primary antibodies**

**used for immunocytochemistry**

| Antibody | Vendor | Cat. No. | Dilution |
| --- | --- | --- | --- |
| Nestin | Santa Cruz | SC-21247 | 1:800 |
| MAP2 | Sigma | M4403 | 1:1000 |
| β III-tubulin | Sigma | T8660 | 1:3000 |
| Lmx1a | Santa Cruz | sc-134990 | 1:100 |
| Nurr1 | Santa Cruz | sc-990 | 1:200 |
| Tyrosine hydroxylase | Millipore | AB152 | 1:1000 |
| Tyrosine hydroxylase | Sigma | T2928 | 1:1000 |
| GIRK2 | Abcam | ab30738 | 1:100 |
| GABA | Sigma | A2052 | 1:1000 |
| α-Synuclein | BD | 610787 | 1:400 |
| LC3 | Nanotools | 0231-100/LC3-5F10 | 1:200 |
| LAMP2 | Abcam | Ab25631 | 1:100 |
