# Supplementary figures and images for: Elevated α-synuclein caused by SNCA gene triplication impairs neuronal differentiation and maturation in Parkinson's patient-derived induced pluripotent stem cells
Source: Cell Death Dis. 2015 Nov 26;6(11):e1994–. doi: 10.1038/cddis.2015.318 (PMC4670926; doi:10.1038/cddis.2015.318)

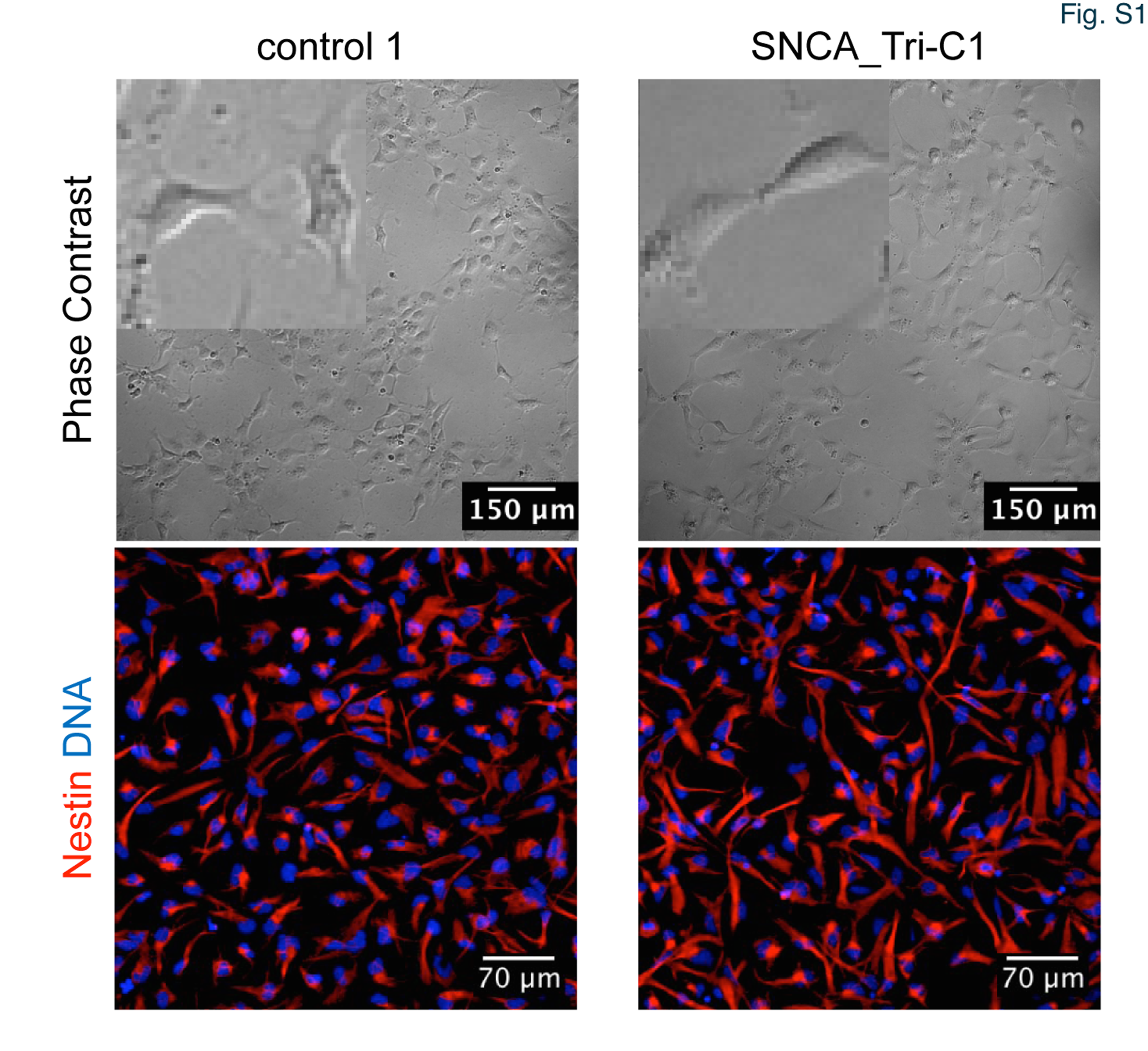

Supplement: Supplementary Figure S1 [file cddis2015318x4.tif]

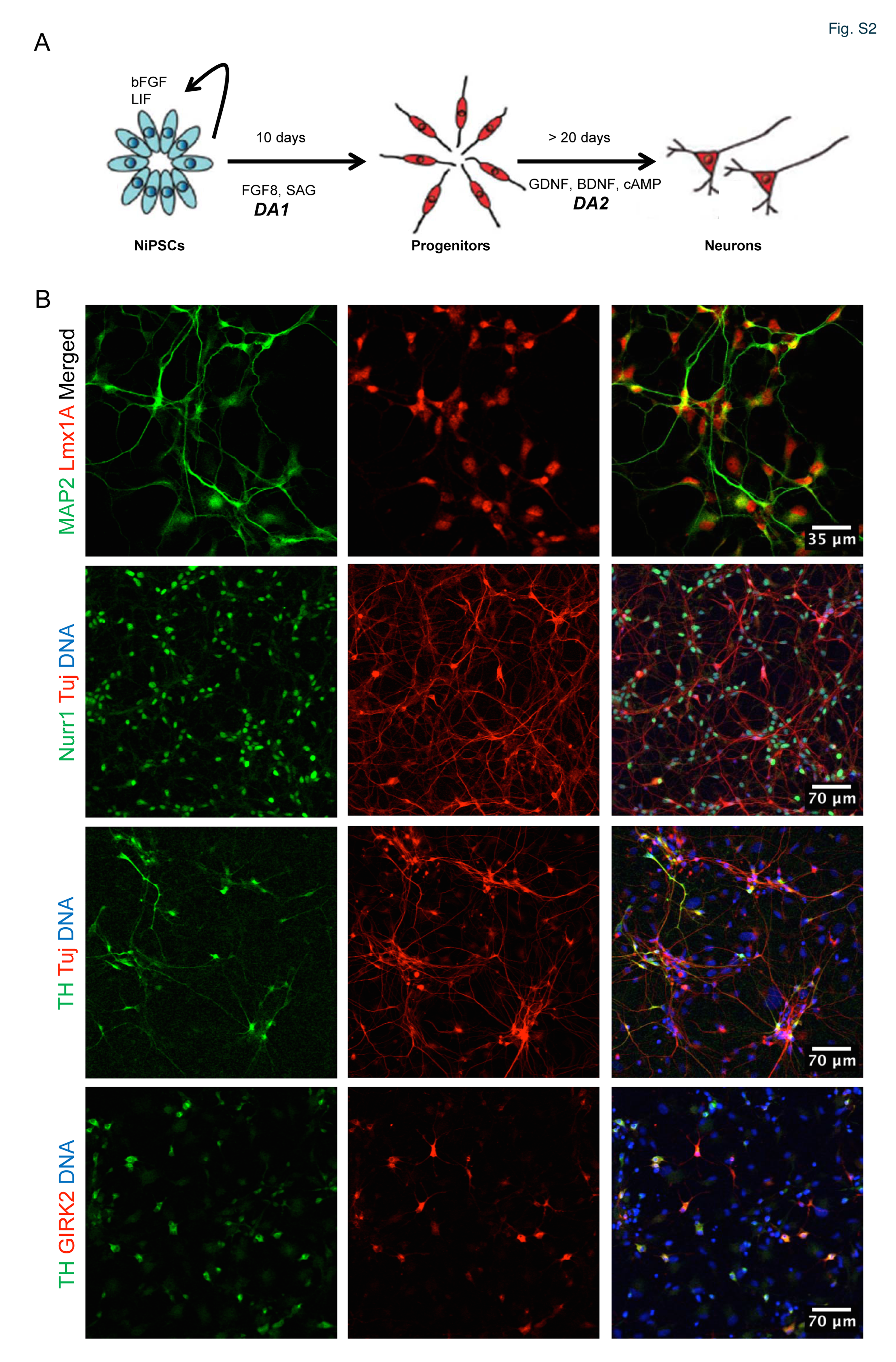

Supplement: Supplementary Figure S2 [file cddis2015318x5.tif]

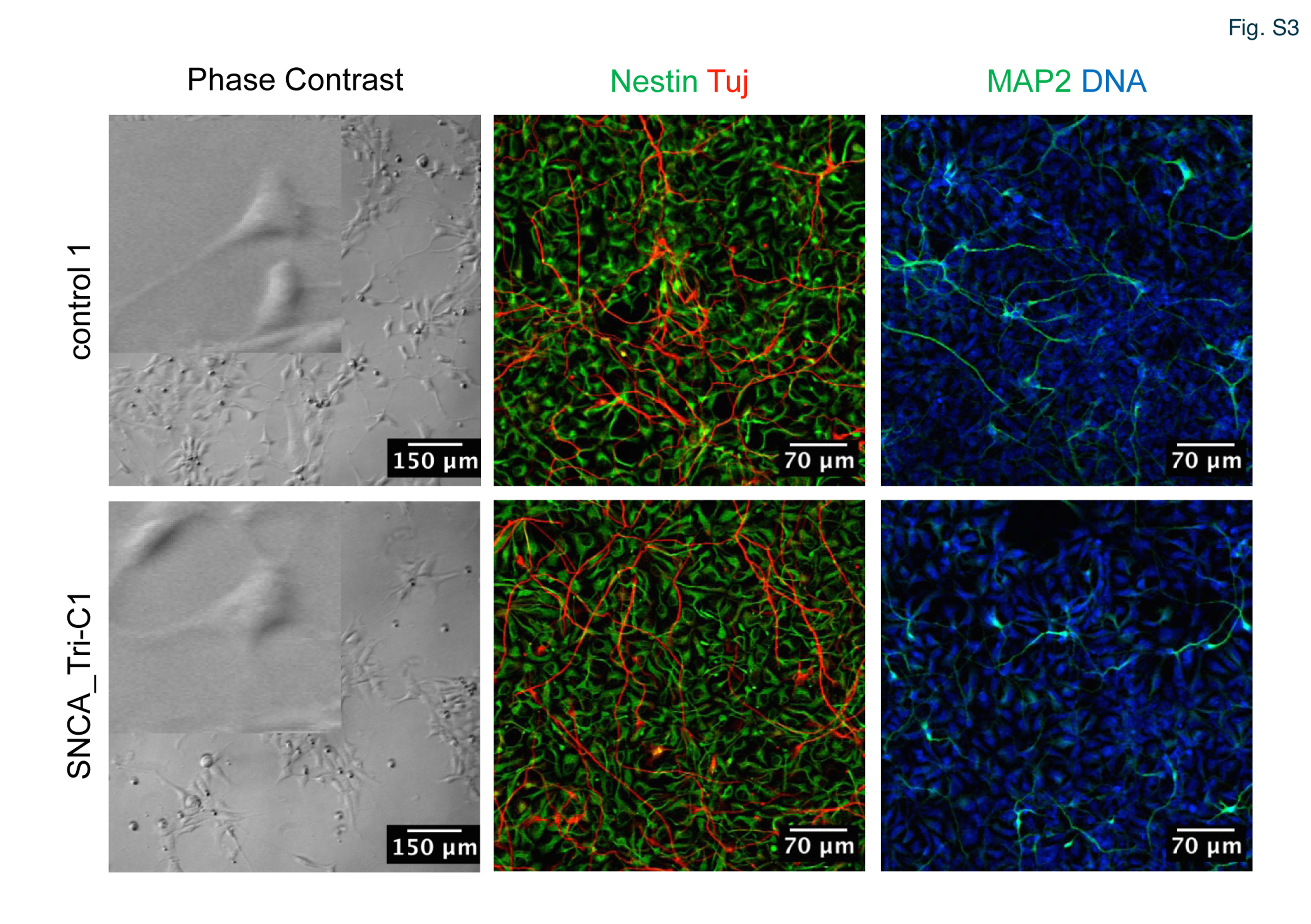

Supplement: Supplementary Figure S3 [file cddis2015318x6.tif]

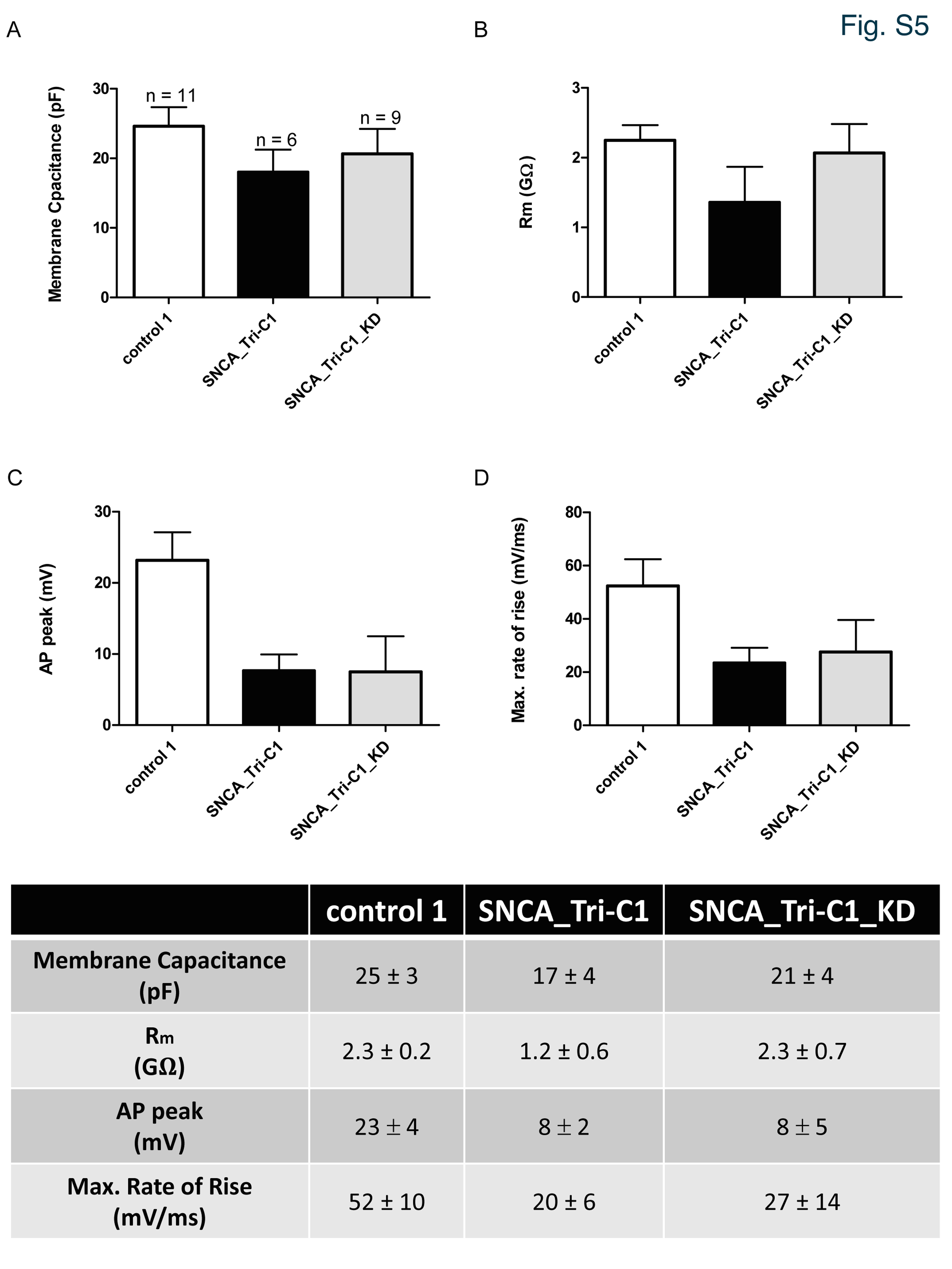

Supplement: Supplementary Figure S4 [file cddis2015318x7.tif]

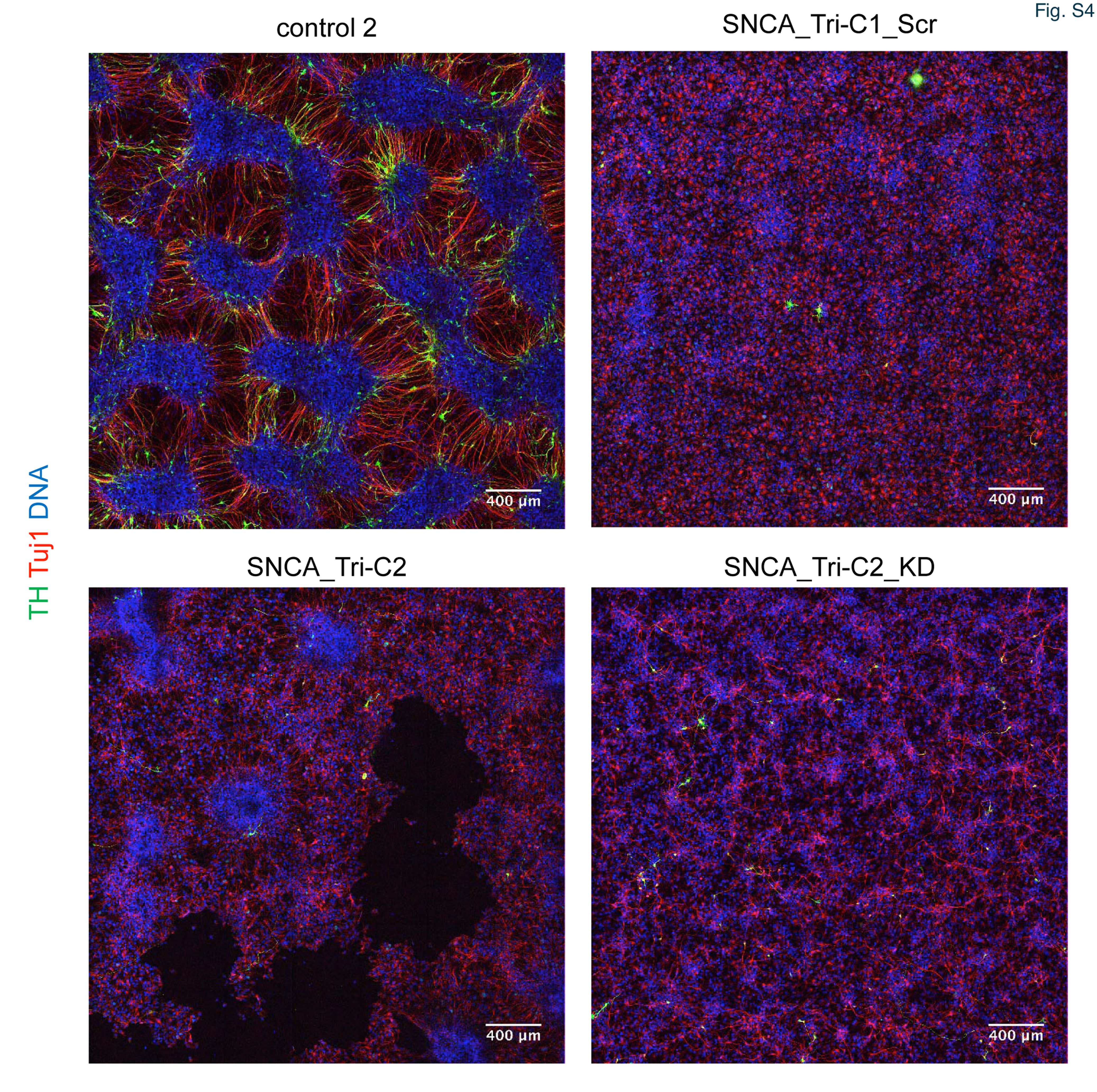

Supplement: Supplementary Figure S5 [file cddis2015318x8.tif]

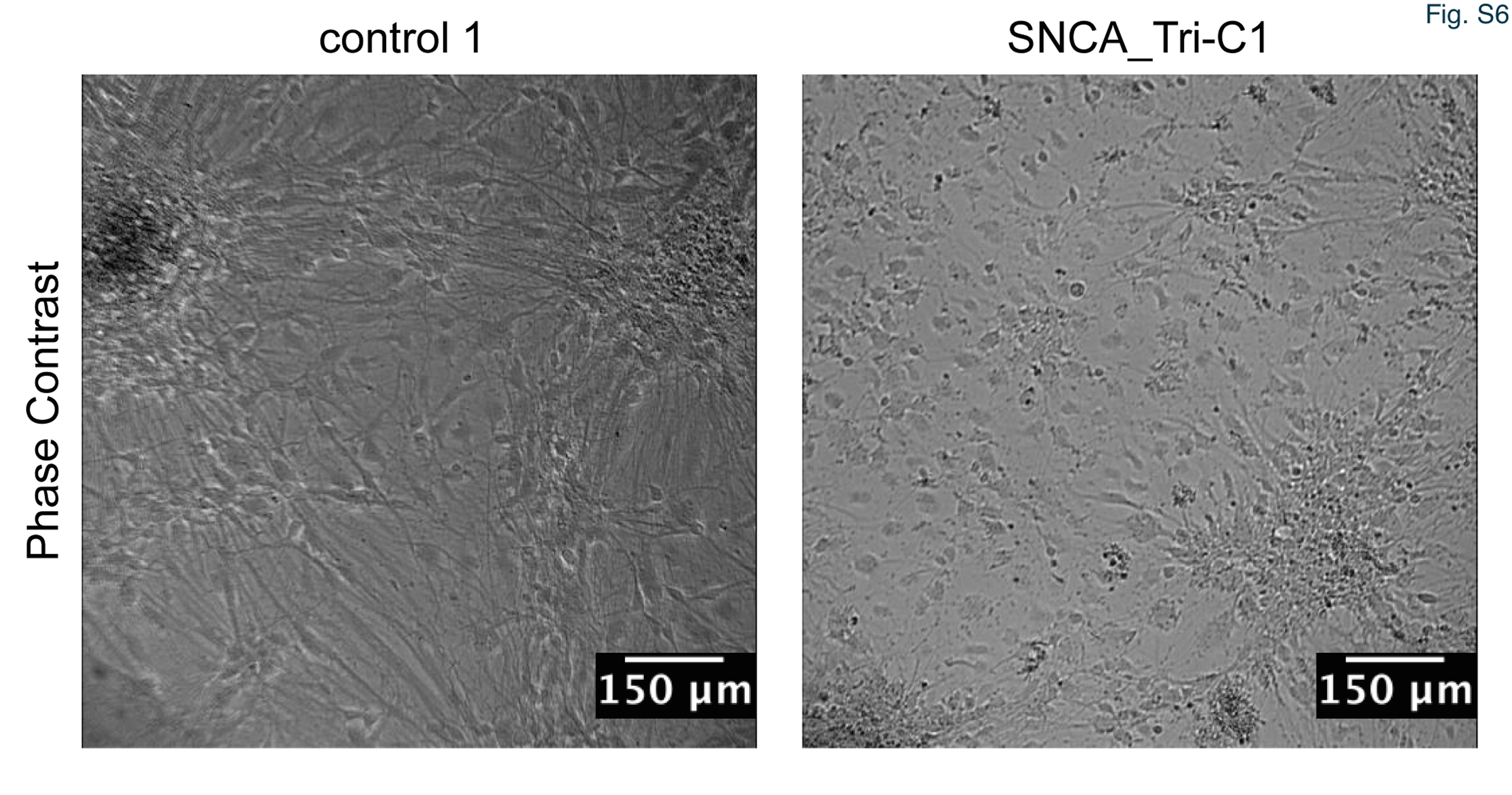

Supplement: Supplementary Figure S6 [file cddis2015318x9.tif]
