# Supplementary material for: Elevated α-synuclein caused by SNCA gene triplication impairs neuronal differentiation and maturation in Parkinson's patient-derived induced pluripotent stem cells
Source: Cell Death Dis. 2015 Nov 26;6(11):e1994–. doi: 10.1038/cddis.2015.318 (PMC4670926; doi:10.1038/cddis.2015.318)
Supplement: Supplementary Information [file cddis2015318x11.docx]

**Supplementary Figure Legends**

**Supplementary Figure S1.** **Characterization of Control and SNCA_Tri NiPSCs.** Phase contrast (upper panels) and immunofluorescence (bottom panels) images. All cells were positive for the neural progenitor marker Nestin (red). Nuclei were counterstained with DRAQ5 (blue). Insets in phase contrast images are 5× digital zooms from the surrounding images.

**Supplementary Figure S2.** **Differentiation of the NiPSC lines.** (**A**) Schematic representation of the 30-day differentiation protocol to generate DAn: 10-day culture with FGF8 and SAG (DA1 Medium) for midbrain specification and greater than 20 days in the presence of BDNF, GDNF and cAMP (DA2 medium) for dopaminergic maturation. (**B**) Immunofluorescence of the differentiated NiPSCs (Control line) showing expression of Lmx1a, MAP2, Tuj1, Nurr1, TH and GIRK2, (DNA by DRAQ5 staining), confirming the generation of DAn.

**Supplementary Figure S3.** **Characterization of Control and SNCA_Tri after DA1 differentiation.** Phase contrast (left) and immunofluorescence (center and right) images. Neural progenitor marker Nestin (center - green); early neuronal markers βIII-Tubulin (center - red) and MAP2 (right - green); nuclei (DRAQ5 positive, blue, right). Insets in phase contrast images are 5× digital zooms.

**Supplementary Figure S4.** **Yield of** **dopaminergic neurons after DA2 differentiation.** TH immunofluorescence (data of Fig. 2C) for the extra cell lines used for dopaminergic quantification.

**Supplementary Figure S5.** **Electrophysiological characterization of differentiated NiPSCs.** Passive and active membrane properties obtained from cells that generated APs. The number of cells analyzed in each group is indicated in A. Cell membrane capacitance **(A)** and input resistance (R_m_) **(B)** were estimated from integrals of current transients that were elicited by small hyperpolarizing voltage pulses (10 mV) from a holding potential of -70 mV after compensating the pipette’s capacitance in cell-attached mode. **(C)** Peak amplitudes of APs elicited by 100 ms sustained current injection. **(D)** Maximum rates of rise estimated by measuring the peak of the first derivative of the membrane potential. The table below the figure presents the values obtained for each of the evaluated parameters.

**Supplementary Figure S6. Long-term differentiation of NiPSCs from Control and SNCA_Tri.** Phase contrast images of live NiSPC Control (left) and SNCA_Tri (right) lines differentiated for 64 days.

**Supplementary Movie 1. DA2-differentiation of Control and SNCA_Tri lines.** Control and patient cells plated in poly-L-lysine plus laminin-coated µ-dishes at 3.1·10^2^ cells/mm^2^. Phase contrast images were taken at the indicated days of DA2 differentiation and in the same region of the dish. Images were compiled with Fiji and play at 1 frame/sec.
